# Pentafluorobenzene end-group as a versatile handle for *para* fluoro “click” functionalization of polythiophenes†
†Electronic supplementary information (ESI) available: Experimental details, MALDI-TOF, ^1^H and ^19^F NMR, UV-visible spectroscopy, and photoluminescence spectroscopy results. See DOI: 10.1039/c6sc04427a



**DOI:** 10.1039/c6sc04427a

**Published:** 2016-12-15

**Authors:** Pierre Boufflet, Abby Casey, Yiren Xia, Paul N. Stavrinou, Martin Heeney

**Affiliations:** a Dept. Chemistry and Centre for Plastic Electronics , Imperial College London , Exhibition Rd , London , SW7 2AZ , UK . Email: m.heeney@imperial.ac.uk; b Dept. Physics and Centre for Plastic Electronics , Imperial College London , Exhibition Rd , London , SW7 2AZ , UK; c Dept. of Engineering Science , University of Oxford , Parks Road , Oxford OX1 3PJ , UK

## Abstract

A convenient method of introducing pentafluorobenzene (PFB) as a single end-group in polythiophene derivatives is reported *via in situ* quenching of the polymerization. We demonstrate that the PFB-group is a particularly useful end-group due to its ability to undergo fast nucleophilic aromatic substitutions. Using this molecular handle, we are able to quantitatively tether a variety of common nucleophiles to the polythiophene backbone. The mild conditions required for the reaction allows sensitive functional moieties, such as biotin or a cross-linkable trimethoxysilane, to be introduced as end-groups. The high yield enabled the formation of a diblock rod-coil polymer from equimolar reactants under transition metal-free conditions at room temperature. We further demonstrate that water soluble polythiophenes end-capped with PFB can be prepared *via* the hydrolysis of an ester precursor, and that such polymers are amenable to functionalization under aqueous conditions.

## Abstract 2

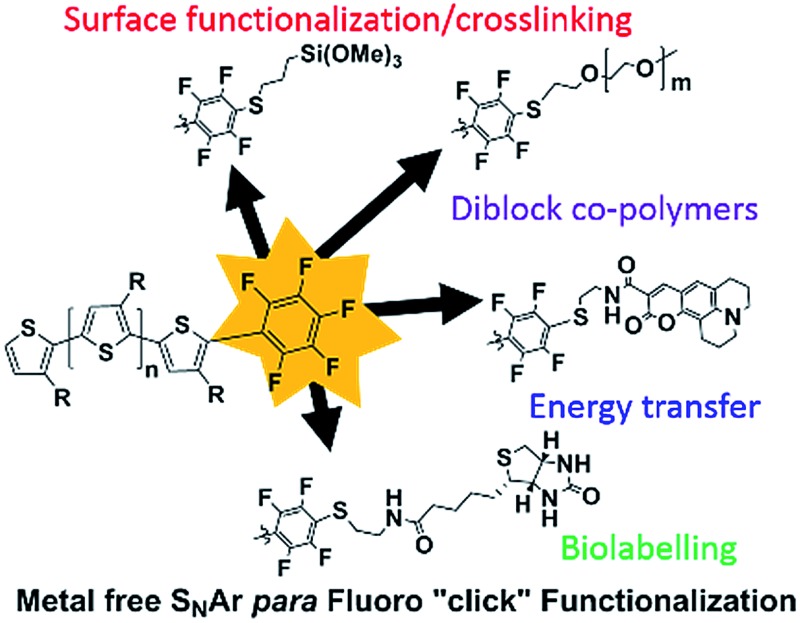
Inclusion of a perfluorophenyl endgroup enables the ready functionalization of water soluble polythiophenes under mild conditions.

## Introduction

Polythiophenes are among the most promising materials for applications in organic electronics.^1–3^ Their end-group functionalization has also garnered much interest since it leads to the attractive prospect of having a molecular handle for self-assembly, surface modification, biomarking, charge transfer and for the growth of block copolymers.^4–6^

Three major end-group functionalization techniques have been explored to date for polythiophene systems. External initiation of the Kumada catalyst-transfer polymerization (KCTP) is reported to offer excellent control over the end-group composition.^7–13^ However, this method does suffer from a few limitations, such as the requirement to use air-sensitive Ni(0) to form the initiator and some limitations on the choice of aryl (end)group used for initiator formation.^9,14,15^

*In situ* end-capping, on the other hand, opens the door for integration of a wide variety of functional groups onto the end of polythiophene chains. This method has been investigated by many groups in recent years, but can suffer from poor control of the degree of end-functionalization, with mixtures of incomplete mono-, and di-functionalization being commonly reported.^16–22^ Both externally initiated and *in situ* end-capping methods additionally require the protection of any functional groups that may poison the catalyst or be sensitive to the strongly basic conditions present during the KCTP.^10,11^

The third choice is post-polymerization modification of pre-formed polythiophenes. This has been explored *via* the reactivity of the Br and H end-groups inherently formed after traditional KCTP. However the scope of such reactions has so far been limited to transition metal-catalyzed cross-coupling reactions or quenching of metallated poly(3-hexylthiophene) (P3HT) with the desired electrophile.^4,23–25^

**Fig. 1:**
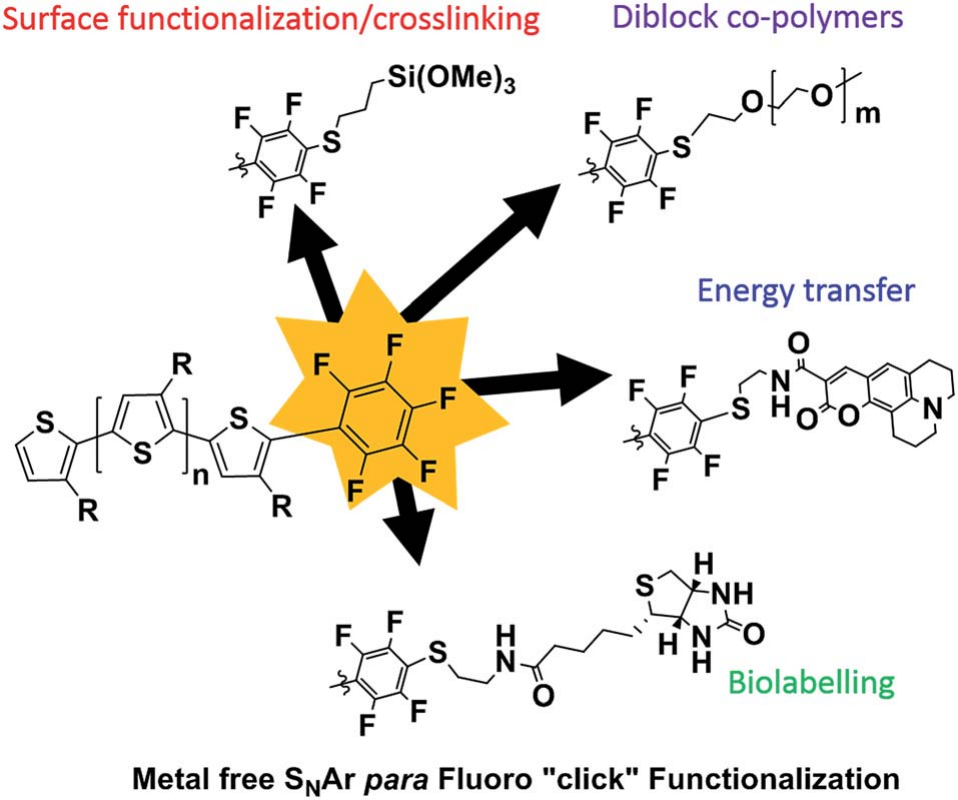
Proposed post-polymerisation of pentafluorophenyl endcapped poly(3-alkyl)thiophene with a range of functionalised thiols.

In this work we explore the *in situ* approach to singly end-cap poly(3-octylthiophene) (P3OT) with pentafluorobenzene (PFB), a group that is sufficiently stable towards basic conditions to avoid the need for protecting groups, but is nevertheless reactive enough to be functionalized directly with a range of nucleophiles post-polymerization without further modification (Fig. 1). Although the nucleophilic aromatic substitution of PFB with a range of nucleophiles under mild conditions has long been recognized, it has received comparatively little attention as a useful medium for fast and near quantitative “click” reactions.^26–31^ It's potential has been utilized in polymer systems, where non-conjugated polymers containing PFB side-groups were shown to undergo nucleophilic aromatic displacement with good nucleophiles in near quantitative yields, a reaction termed *para* fluoro “click”.^32–39^ To the best of our knowledge, only one example of functional PFB incorporation in a conjugated polymer system has been reported.^40^ In that study, the PFB group was introduced onto short oligomers of 3-hexylthiophene post-polymerization *via* Suzuki coupling, and the resulting polymer was used for TiO_2_ nanoparticle functionalization. We note that the PFB-functionalized P3HT synthesized in the aforementioned study was only partially characterized however, and the ^19^F NMR data presented is somewhat different to the typical chemical shift and multiplicities observed for aryl-PFB species (*vide infra*).

By combining the PFB reactivity with the advantages of *in situ* end-capping, we wished to broaden the scope for post-polymerization modification without the need for specific initiators, protecting groups or expensive/hazardous coupling reagents.

In this report, we demonstrate that the PFB can be incorporated as an end-group in poly(3-octyl)thiophene in good fidelity by an optimized *in situ* quenching procedure. We show that nucleophilic aromatic substitution (S_N_Ar) can occur on the PFB-end-capped P3OT with common nucleophiles such as thiols, amines, and alcohols, all under mild conditions and quantitative yield. The method is also amenable to the difficult coupling of two polymers, to form a diblock copolymer, in this case using a thiol end-capped PEG. To further highlight the utility of this approach, several sensitive functional groups were incorporated onto the polymer end-group (Fig. 1). For example biotin, a molecule extensively studied and used for its strong binding to the protein avidin, was tethered to the mono-functionalized polymer *via* a thiol linker, opening the door for further biomarkers to be investigated.^41–43^

The potential for Förster Resonance Energy Transfer (FRET) between the polythiophene and dye molecule is an attractive prospect, but relies heavily on the close special proximity of donor and acceptor moieties, as well as the appropriate spectral overlap of absorption and emission.^44^ Blending potentially leads to issues of phase segregation and self-quenching, so chemically binding the two components is a useful approach to assure that the donor and acceptor are never too distant from each other, and the FRET efficiency is as high as possible. The reactivity of the PFB group towards alkyl thiols under very mild conditions was therefore used to attach an easily synthesized thiolated derivative of Coumarin 343. The resulting system, although only possessing slightly complimentary absorptions from the dye and P3OT components in the solid state, demonstrates the proof-of-concept energy transfer from the dye to the P3OT. We also demonstrate that moisture sensitive trimethoxysilane groups can also be readily incorporated *via* the reaction of commercially available reagent (3-mercaptopropyl)trimethoxysilane. The resulting alkoxysilane end group of the polymer can be utilized to form an intractable thin film on the surface of glass.

Finally we report an endcapped ester functionalized polythiophene which is soluble in polar aprotic solvents, allowing for much shorter S_N_Ar reaction times. Saponification of the ester functionalities afforded a water soluble polythiophene, which could be end functionalized in a mixture of water and DMF, potentially facilitating reactions under biological conditions.^45^

## Results and discussion

### Optimization of the end-capping procedure

We opted to use matrix-assisted laser desorption–ionization time-of-flight (MALDI-TOF) mass spectrometry for the optimization of the end-capping conditions since both ^1^H and ^19^F NMR spectroscopy could not distinguish between mono- and di-end-capped polymer chains. This led to the need for a different alkyl-chain length than the more widely studied hexyl (in P3HT) since a 3-hexylthiophene repeat unit has a near identical exact mass to the PFB group (166.06 gmol^–1^ and 166.99 gmol^–1^ respectively). End-group analysis of the PFB end-capped P3HT would have required the use of deconvolution software and modeling, as was elegantly used by Pickel *et al.*, an approach we deemed beyond the scope of this study.^17,18^ We, therefore, opted for the 3-octylthiophene repeat unit, since its difference of 28 gmol^–1^ with the PFB end-group is sufficient to be satisfactorily resolved by MALDI-TOF.

**Scheme 1:**
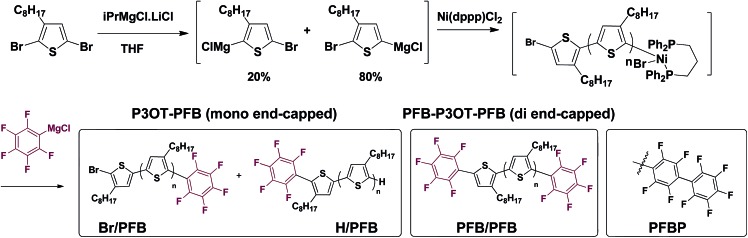
*In situ* mono- and di-endcapping of P3OT using pentafluorophenylmagnesium chloride (PFB-MgCl).

Typical *in situ* end-capping procedures of GRIM polymerizations involve adding a desired end-capping Grignard reagent to an ongoing polymerization reaction.^16–22^ The polymerization was performed under the usual procedures (Scheme 1). Briefly, a solution of 2,5-dibromo-3-octylthiophene was treated with isopropylmagnesium chloride-lithium chloride complex for 1 h at room temperature, after which the Ni(dppp)Cl_2_ catalyst was added in one portion to initiate the polymerization. The end-capping Grignard reagent was then added dropwise to the polymerization at either room temperature or 0 °C, and the reaction quenched with 5 M HCl at the desired time interval.

An excess of the end-capper, relative to the catalyst (4–20 times), is usually added to ensure maximal end-capping, however the relative extent of mono- and di-end-capped polymer varies significantly according to the nature of the end-capping reagent. To the best of our knowledge, only vinyl- or allyl-based end-capping reagents lead to near-exclusive mono-end-capped polymer chains, with the exception of 3-[bis(trimethylsilyl)amino]phenylmagnesium chloride reported by McCullough *et al.* ^22^ Mindful that an excess of pentafluorophenylmagnesium chloride (PFB-MgCl) would not only lead to di-end-capping, but potentially lead to the formation of perfluorinated biphenyl (PFBP) end-groups as a result of nucleophilic aromatic substitution on the PFB end-group by PFB-MgCl (Scheme 1),^46,47^ we performed our initial end-capping procedure using a 6-fold excess of PFB-MgCl. The progress of the end-capping was monitored through MALDI-TOF analysis of aliquots at time intervals of 2 and 70 minutes to assess the influence of prolonged reaction times on end-capping.

The results, shown in Fig. S1† and summarized in Table 1, clearly illustrate the fast rate of the end-capping reaction. Indeed, after only 2 minutes, a majority (*ca.* 64%) of the P3OT chains are mono-endcapped with PFB groups, either with or without a bromine atom on the other chain end (Br/PFB and H/PFB respectively). The remaining polymer chains are terminated predominantly with bromine and hydrogen atoms on opposite ends (Br/H), and a small minority has hydrogen or bromine on both ends (H/H and Br/Br respectively). Also apparent is a trace amount of doubly PFB end-capped chains (PFB/PFB). The aliquot taken after 70 minutes shows a substantial change in the end-group composition, with PFB/PFB end-groups being the most abundant, followed by H/PFB. As has been suggested by other groups,^16–18,22^ this can be rationalized by considering that Br/PFB and Br/H chain ends can respectively be readily converted to PFB/PFB and PFB/H with the residual PFB-MgCl and Ni(dppp). The reason for the slower conversion of Br/H to H/PFB as compared to Br/PFB to PFB/PFB is unclear, but likely arises from the higher concentration of Br/PFB compared to Br/H. The formation of PFBP end-groups is also observed, indicating that the nucleophilic aromatic displacement can occur at room temperature.

**Table 1:** Summary of end-capping procedure optimization

Entry	PFB-MgCl^*a*^ (eq.)	Temperature	2 minutes^*b*^			70 minutes^*b*^		
			Mono-PFB	Di-PFB	PFBP?	Mono-PFB	Di-PFB	PFBP?
1	0.3	RT	64%	3%	Yes	37%	30%	Yes
2	0.3	0 °C	64%	0%	No	66%	4%	No
3	0.1	0 °C	49%	0%	No	65%	6%	No

^*a*^A 0.5 M solution of pentafluorophenylmagnesium chloride (PFB-MgCl) freshly prepared from iodopentafluorobenzene and *n*-butylmagnesium chloride at –78 °C.

^*b*^Approximate composition based on matrix-assisted laser desorption–ionization time-of-flight (MALDI-TOF) peak intensities.

In an effort to suppress the formation of PFBP end-groups and the double PFB end-capping, the procedure was performed at 0 °C (Fig. S2†). After 2 minutes, 64% of the chain ends are singly end-capped with PFB, as observed at room temperature. Gratifyingly, the PFB/PFB terminated P3OT polymer chains are absent from the MALDI spectrum at 2 min, although they appear as very minor products (*ca.* 4%) after 70 minutes, without a noticeable change in the Br/PFB and H/PFB content (64%). Reducing the equivalents of PFB-MgCl used to quench the polymerization to a 2-fold excess compared to the catalyst loading (Fig. S3†) results in a significant reduction in PFB incorporation after 2 minutes (49%). Presumably due to their high relative concentration, Br/H polymer chains are converted to H/PFB after 70 minutes, bringing the total mono-PFB end-capped P3OT up to 65%. Unfortunately, Br/PFB chains are also partly converted to PFB/PFB (6%).

The optimal reaction conditions for single end-capping were found to occur after 10 minutes with a 2-fold excess of PFB-MgCl. Mono-PFB end-capping reached 70%, and PFB/PFB terminated P3OT remained undetected by MALDI-TOF.

**Fig. 2:**
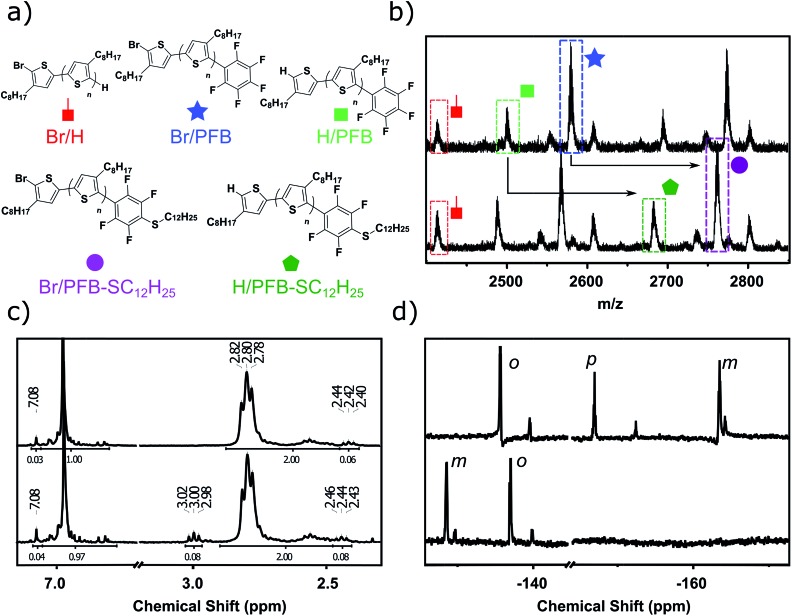
(a) Assigned structures and respective end-groups. (b) Assigned matrix-assisted laser desorption ionization (MALDI) mass spectrometry of P3OT–PFB (top) and P3OT–PFB–SC_12_H_25_ (bottom). (c) Selected aromatic and methylene regions of ^1^H NMR spectra of P3OT–PFB (top) and P3OT–PFB–SC_12_H_25_ (bottom) showing the new triplet at 3.00 ppm assigned to the PFB–S–CH_2_– group. (d) ^19^F NMR spectra of P3OT–PFB (top) and P3OT–PFB–SC_12_H_25_ (bottom), with *ortho* (*o*), *meta* (*m*), and *para* (*p*) positions assigned.

Fig. 2d shows the ^19^F Nuclear Magnetic Resonance (NMR) spectroscopy of singly PFB end-capped P3OT, referred to henceforth as P3OT–PFB. Three major signals are present at –137.8 ppm, –153.5 ppm and –161.7 ppm, assigned to *ortho*, *para* and *meta* positions on the PFB moiety respectively.^37,38,48^ Weaker resonances at –139.8 ppm, –156.2 ppm, and –162.1 ppm are attributed to the PFB moiety when tethered to a non-head-to-tail thiophene chain end.

The ^1^H NMR of P3OT–PFB (Fig. 2c) also suggests successful incorporation of the PFB end-group, with a resonance appearing downfield compared to other aromatic signals, at 7.08 ppm. This is consistent with the electron-withdrawing effect of the PFB group. Based on the shift observed after reacting the PFB group with alkylthiols (*vide infra*) as well as the behavior of 2,5-bis(perfluorophenyl)-3-methylthiophene,^49^ the methylene protons on the terminal thiophene-PFB moiety are tentatively assigned to the triplet at 2.42 ppm. The reduced chemical shift compared to other end-groups is likely a result of a through-space shielding effect of the protons by fluorine atoms.^50^ Integration of the methylene region of the NMR indicates an 86% head-to-tail regioregularly.^51^ This low value is predominantly attributed to end-group effects as a result of the low molecular weight of the samples. Indeed, gel-permeation chromatography (GPC) in chlorobenzene at 80 °C indicates that entry 3 exhibits a number average molecular weight (*M*_n_) of 5.4 kg mol^–1^, and a dispersity index (*Đ*) of 1.3. MALDI-TOF of the same sample exhibits a maximum abundance for polymer chains of *ca.* 2.4 kg mol^–1^ (*M*_n_ 2.9 kg mol^–1^). This discrepancy between measured GPC and MALDI-TOF molecular weights at *M*_n_ < 20 kg mol^–1^ has been previously documented for polythiophenes, and is related both to the rigid-rod behavior of regioregular poly(3-alkythiophenes) compared to the coil behavior of the polystyrene standards used in GPC, and the difficulties in ionizing higher molecular weight polymer chains.^52–55^

UV-vis spectra of P3OT and P3OT–PFB (Fig. S4†) show that, in solution, the PFB group has no obvious effect on the shape or position of the absorption spectrum, nor is there any indication of donor–acceptor type interactions between the polythiophene backbone and the electron deficient PFB end-group. In the thin-film however, slight differences are observed between the two polymers, with P3OT–PFB exhibiting a 5 nm red-shift in the *λ*_max_ compared to P3OT, as well as a reduction in the high-energy region (400–500 nm) typically attributed to disordered poly(3-alkylthiophenes).^56,57^

### Investigating end-group reactions

In order to probe the reactivity of P3OT–PFB a test reaction with excess 1-dodecanethiol in THF for 12 h at 70 °C in the presence of K_2_CO_3_ was performed. In the case of a singly substituted pentafluorobenzene, nucleophilic attack usually occurs at the *para*-position, with some substituents leading to *ortho*-substitution.^58^ Under the conditions described above, the nucleophilic aromatic substitution on P3OT–PFB proceeded smoothly, with the new mono-substituted product clearly observed by MALDI (Fig. 2a and b). The reaction proceeded regioselectively at the *para*-position, as demonstrated by the two double doublet signals at –134.3 ppm and –138.5 ppm observed by ^19^F NMR (Fig. 2d). Despite the large excess of thiol nucleophile present in the reaction mixture, and the prolonged reaction time, further nucleophilic displacements on the PFB ring were not observed. It is also noteworthy that the small amount of Br/H P3OT present (*i.e.* non PFB endcapped) is inert under these conditions (Fig. 2b). To explore the scope of this PFB end-group, the reaction with a variety of thiol, amine and alcohol nucleophiles under similar conditions was explored, along with a selection of functional molecules (Table 2).

**Table 2:** Scope of reaction of P3OT–PFB with nucleophiles

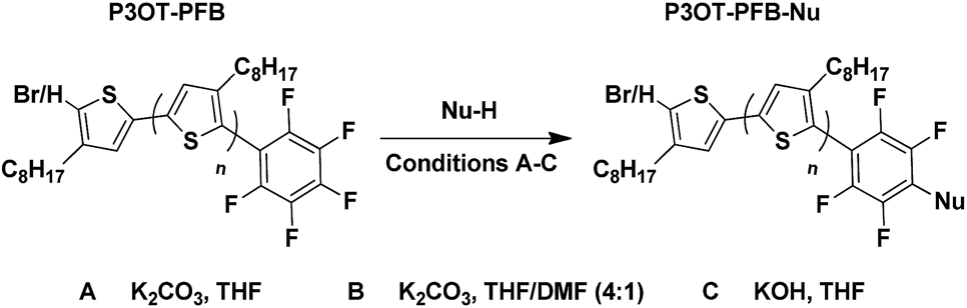				
Nucleophile	Conditions^*a*^	Temp.	Time	Conversion^*b*^
C_12_H_25_–SH	A	70 °C	12 h	Full
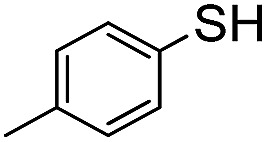	A	RT	24 h	None
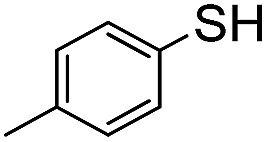	A	70 °C	12 h	Full
C_8_H_17_–NH_2_	A	70 °C	48 h	Full
C_8_H_17_–NH_2_	B	70 °C	12 h	Full
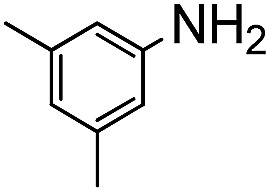	A	70 °C	48 h	None
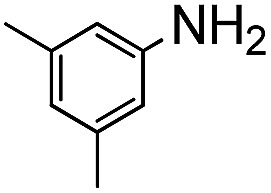	B	70 °C	48 h	None
C_5_H_11_–OH	A	70 °C	48 h	None
C_5_H_11_–OH	B	70 °C	48 h	None
C_5_H_11_–OH	C	70 °C	12 h	Full (disub.)
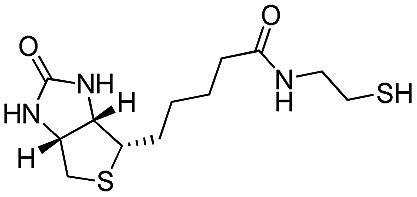	A	70 °C	12 h	Full
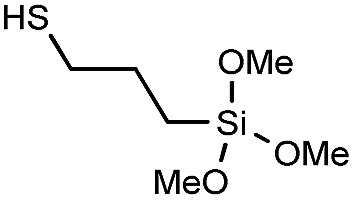	A^*c*^	70 °C	12 h	Full
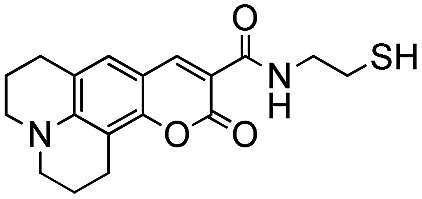	A	RT	48 h	Full

^*a*^Reaction conditions: 0.02 μM scale (relative to PFB group), 10 eq. nucleophile, 15 eq. base in a sealed pressure resistant MW vial.

^*b*^Conversion as evident from MALDI-TOF and ^19^F NMR.

^*c*^Anhydrous conditions.

Conversion of the PFB group was assessed using a combination of ^19^F NMR and MALDI-TOF. The reaction with alkylthiols proceeds smoothly under the conditions outlined above, and can even be performed at room temperature, albeit with longer reactions times. Thus, sensitive functional molecules such as the Coumarin 343 derivative used here can be tethered to the polythiophene *via* the PFB end-group in quantitative yield after reaction for 48 h.

The weaker thiol nucleophile *p*-thiocresol on the other hand showed no conversion after 24 h at room temperature, but heating to 70 °C led to complete conversion after 12 h. The reaction with *n*-octylamine was found to be slower than with alkylthiols, and required prolonged heating times (48 h) or the addition of a polar co-solvent (DMF) to obtain full conversion in 12 h. Unlike its alkylamine counterpart, the aniline-based nucleophile attempted (3,5-dimethylaniline) failed to substitute any of the fluorine atoms, likely due to the lower basicity of anilines compared to alkylamines. Interestingly, while pentanol did not lead to substitution using K_2_CO_3_ as a base, even in the presence of DMF, changing the base to KOH produced a majority of the disubstituted P3OT–PFB under the standard reaction conditions, with the second alkoxy group being directed to the *meta*-position relative to the first alkoxy group (as determined by three ^19^F NMR signals: *δ* (ppm) –139.98 (dd, *J* = 9, 23 Hz, 1F), –150.38 (d, *J* = 9 Hz, 1F), –158.25 (d, *J* = 22 Hz, 1F)). Wiehe *et al.* recently reported analogous reactivity of pendant PFB groups on porphyrin moieties, finding that using KOH as a base was key in obtaining high yields.^59^

The biotin–avidin complex is one of the most widely studied biological systems for uses in sensors and targeted therapy, in part due to their very high binding affinity.^43,60^ Surface modification, fluorescent and luminescent sensing, as well as *in situ* sensing through bilayer organic field effect transistor devices have also used the biotin–avidin system.^42,60–64^ The biotin moiety has also been previously tethered to polythiophene backbones *via* side-chain functionalisation.^65–67^

As a proof of concept for the tethering of biomolecules to P3OT using the PFB molecular handle, we synthesized a previously reported biotinylated thiol derivative^41^ and reacted it with P3OT–PFB under the same conditions as other typical thiol nucleophiles. Despite the low solubility of biotin–SH in organic solvents, the reaction proceeded to completion within 12 hours, albeit with a substantial detrimental effect on the solubility of the resulting polymer. Sufficient solubility in CDCl_3_ was obtained for ^1^H NMR analysis only after the addition of DMSO-*d*_6_ as a co-solvent. ^19^F NMR confirmed the *para*-substitution on the PFB group, and signals corresponding to the biotinylated-P3OT were observed by MALDI-TOF, despite being difficult to ionize in the matrix used (terthiophene). This reaction clearly demonstrates the value of the PFB group in reacting with nucleophile-derivatized biomolecules, and therefore biofunctionalisation without the need for multiple additional steps in monomer synthesis, or expensive coupling reagents.

Modifying surfaces and interfaces with charge transporting and/or light absorbing materials is a major motivating factor for end-capping. P3HT has been extensively used for this purpose, with examples ranging from SiO_2_, TiO_2_, ZnO, carbon nanotubes, as well as a range of quantum dots.^4,5^ Different approaches have been explored to achieve this, including initiation of the KCTP from the surface,^12^ tethering an end-functionalized P3HT to a self-assembled monolayer-treated surface *via* “click” reaction,^68^ or end-functionalization of P3HT with a common anchoring group such as pyridyl, siloxane, phosphonate or thiol.^13,18,19,69^

In order to demonstrate the versatility of the PFB group for integrating functional groups, we reacted P3OT–PFB with the cheap and commercially available reagent 3-mercaptopropyl(trimethoxysilane) (3-MPTS) under the typical nucleophilic displacement conditions. The trimethoxysilane group is known to chemically bind to hydroxylated surfaces like silicon dioxide, however it is very sensitive to hydrolysis in the presence of water, particularly in the presence of acid catalyst.^70^ Therefore reports of the incorporation of trialkoxysilanes in conjugated polymer systems are rare,^69,71^ probably due to the incompatibility of the functional group with typical polymerization conditions. Gratifyingly complete incorporation of 3-MPTS was observed under our mild conditions.

**Fig. 3:**
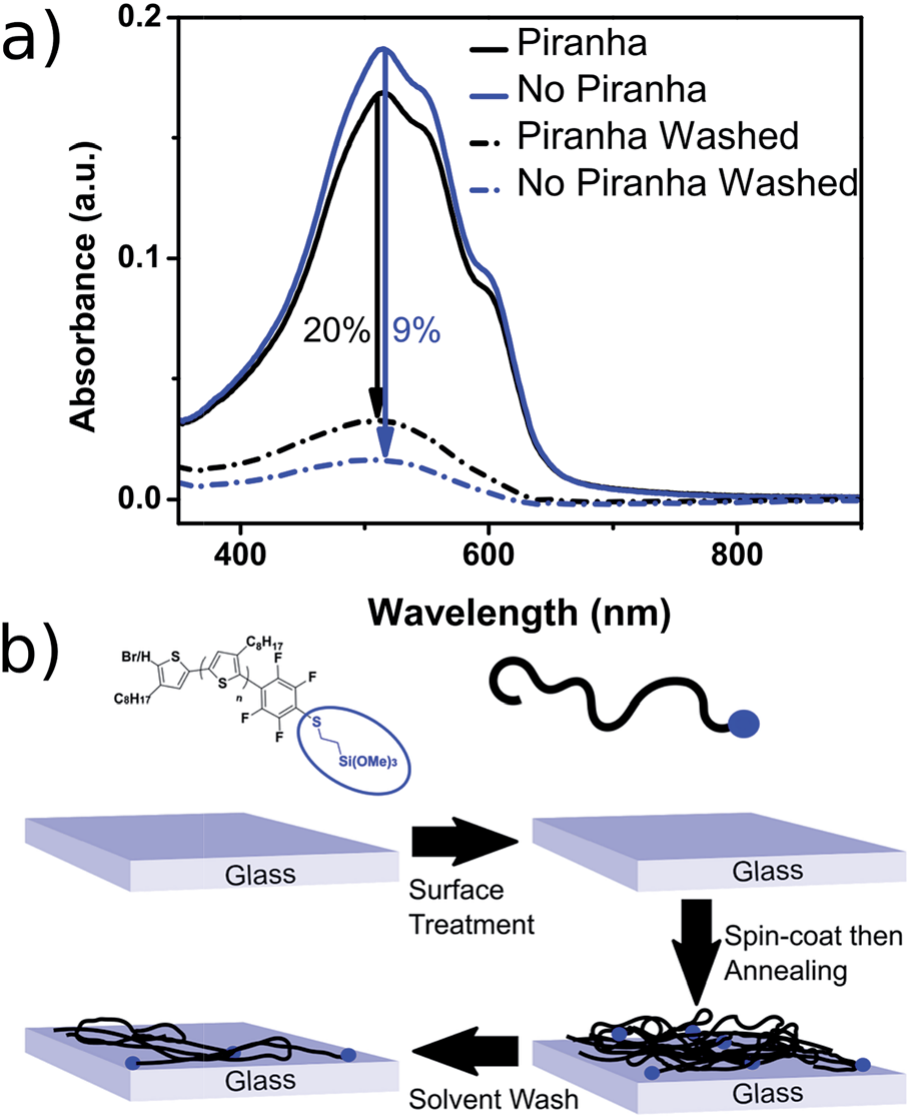
(a) Thin-film UV-visible spectra of P3OT–PFB modified with 3-mercaptopropyl(trimethoxysilane) showing the effect of glass pre-treatment, and a subsequent solvent wash (chloroform), on film retention. Films spun from THF solutions (5 mg mL^–1^) (b) schematic representation of partial polymer retention due to tethering to the glass substrate *via* the siloxane linker (blue dot).

The resulting polymer could easily be used to functionalize glass substrates by spin-coating followed by annealing at 140 °C for 90 minutes. The resulting films were only partially intractable, with slides treated with piranha solution prior to spin-coating retaining up to 20% of the P3OT film after washing with chloroform (see Fig. 3). A possible explanation for the relatively low film retention is that chemical reaction is only possible between one end-group and the glass. As such, polymer chains in the bulk of the film and away from the glass interface are therefore unable to bind and are simply washed away. A control experiment with PFB-endcapped P3OT under the same conditions showed no observable polymer retention (see Fig. S5†). Further optimization of the glass functionalization procedure is required, but the fact that only a single end of the polymer is potentially tethered to the glass is an attractive prospect for engineering of interfaces and monolayers in organic electronic devices.

As already noted, we have also successfully tethered a thiolated Coumarin_343_ derivative to P3OT–PFB thereby providing an attractive system from which to explore the potential energy transfer mechanisms between the dye moiety and the polymer backbone. In this regard, spectra from optical absorption, photoluminescence and time-resolved photoluminescence response have been examined. In dilute THF solutions the constituent materials, the unfunctionalized polymer (P3OT–PFB) and the dye (Coumarin_343_–SH), exhibit featureless absorption profiles with a *λ*_max_ of 440 and 428 nm respectively (see Fig. S6†). The much narrower absorption spectrum of the dye (FWHM: 47 nm), overlaps entirely with that of P3OT–PFB (FWHM: 112 nm). As a result, the functionalized polymer (P3OT–PFB–S–Coumarin_343_) shows an intermediate absorption profile (FWHM: 103 nm); the peak lying closer to that of the dye, at 433 nm, and a shoulder to the red-side, linked to the P3OT–PFB.

Photoluminescence (PL) spectra, recorded from each solution, reveal peak emission wavelengths around 570 nm (Fig. S7†). The PL spectra arising from the functionalized polymer (P3OT–PFB–S–Coumarin_343_) was found to be independent of whether the excitation wavelength fell within the absorption range of the dye (405 nm) or not (465 nm). Further comparisons, with PL spectra from the P3OT–PFB solution, found virtually identical traces; both observations suggesting emission arises exclusively from the P3OT backbone. The recorded transient PL response for both solutions was interpreted using single exponentials, following a deconvolution with the laser pulse (see *e.g.* Fig. S8†). The extracted lifetimes for both solutions ranged between 0.36–0.4 ns (Table S7-1†). The similar values for the lifetimes further supporting the view that all recorded emission, from solutions, originates from the P3OT backbone.

**Fig. 4:**
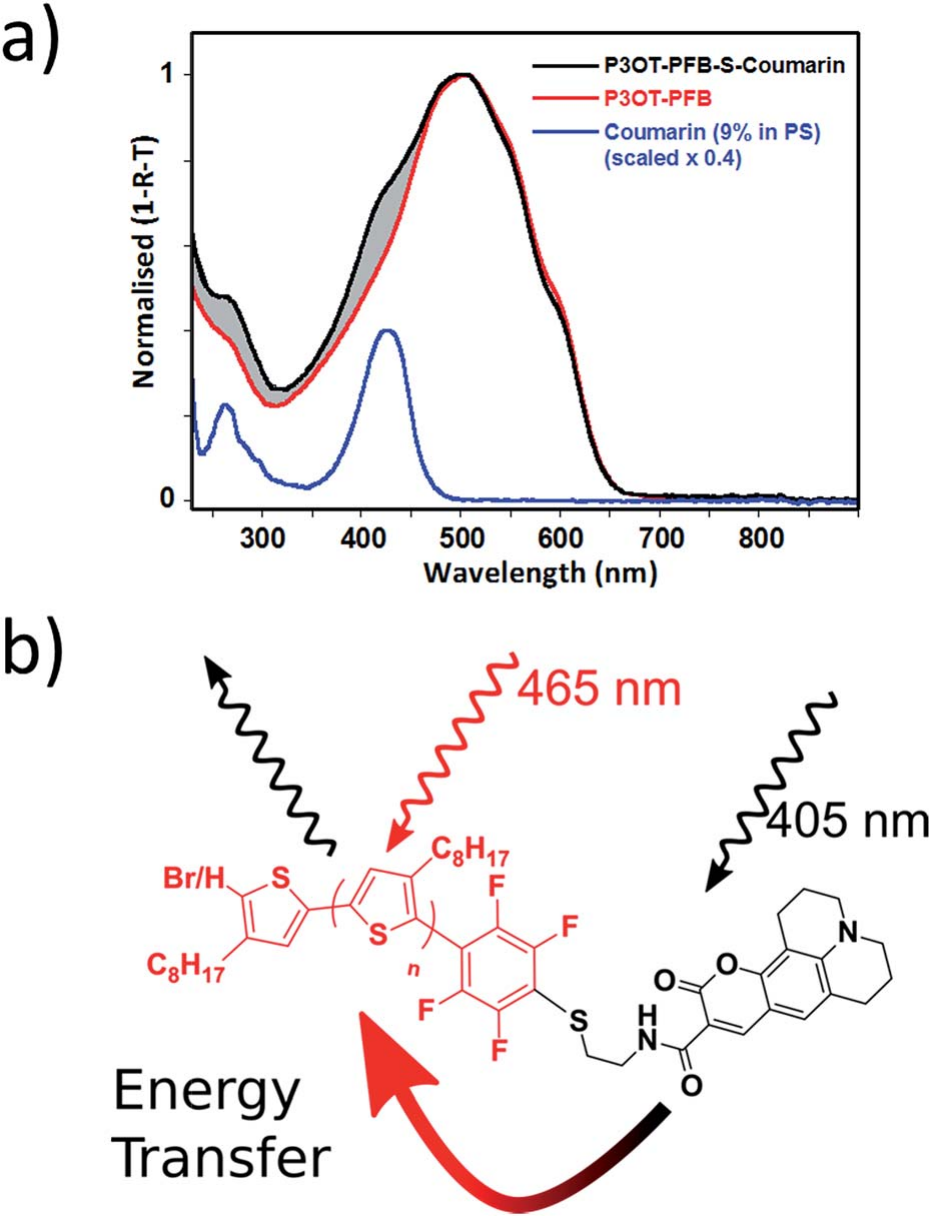
(a) Peak normalized thin-film UV-visible spectra (obtained from recording both specular reflection and transmission) for P3OT–PFB, P3OT–PFB–S–Coumarin_343_, and Coumarin_343_–SH dispersed in a polystyrene matrix (9 wt%). Films spun from THF (5 mg mL^–1^) (b) schematic representation of P3OT–PFB–S–Coumarin_343_ illustrating the likely route of interchain energy transfer and emission from P3OT.

Thin-film absorption spectra of both polymers, along with the Coumarin_343_ dye, were obtained by recording specular transmission (T) and reflection (R) from films spin cast onto fused silica substrates from THF solutions (5 mg mL^–1^) (see Fig. 4). To better represent the dispersion of the Coumarin_343_ moiety within a polymer matrix, and thereby avoid any self-aggregation effects, Coumarin_343_–SH was cast from a solution containing polystyrene of similar molecular weight to P3OT–PFB–S–Coumarin_343_ (*ca.* 2.8 kg mol^–1^). The relative amounts of dye and polystyrene used were calculated from ^1^H NMR integration of P3OT–PFB–S–Coumarin_343_, and were found to be approximately 9 wt% dye relative to the polymer.

In contrast to the absorption spectra of solutions, the broader thin-film spectra of P3OT–PFB is red-shifted, with a peak response *λ*_max_ of 509 nm, and shows evidence of vibronic coupling. Similar behavior, typical of regioregular polythiophenes,^56,72–74^ is also observed in the case of P3OT–PFB–S–Coumarin_343_, which also exhibits a high-energy shoulder around 426 nm, spectrally coincident with the absorption of the pendant dye. The shaded region, between the two normalized spectra of Fig. 4, clearly indicating the additional contribution from dye moiety to the absorption. Despite these differences, the solid-state PL spectra of the two polymers are very similar (see Fig. S9†). Very subtle differences in the vibronic structure are observed, and may indicate slight differences in solid-state order incurred by the bulky dye moiety.^72^ Nevertheless, the position of the peaks observed appear broadly consistent with those from regioregular P3AT derivatives with linear sidechains such as P3HT and P3OT.^74,75^ The similarities in the PL spectra are maintained regardless of the pump wavelength. Crucially, when the excitation pumps both the dye and the P3OT–PFB (at 405 nm), no obvious emission from the dye is detected at lower energy (550 nm), where the emission from Coumarin_343_–SH is typically observed (see Fig. S10†).

In a solid-state format, time-dependent PL measurements (see Fig. S8†) exhibit the expected shortening of the excited state lifetime compared with the solution results (Tables S7-1 and S9-1†). Considering the two polymer systems, the excited state lifetime of the P3OT–PFB–S–Coumarin_343_ system reduces by around 20% compared to the P3OT–PFB. The result holds for both excitation wavelengths, although the absolute values of the (extracted) lifetimes did increase with excitation wavelength. Notice that unlike the studies for solutions, pumping a spin-coated or drop cast sample of the dye at 465 nm, while outside the central absorption peak, does provide some direct excitation of the dye – albeit resulting in a weaker signal (see Fig. S10†). That non-radiative energy transfer mechanisms, such as Förster resonance energy transfer (FRET), are likely to be at play is evident from the consistent reduction of the lifetime for the polymer + dye system. The detailed nature of the processes, however, is not easily ascertained. If one considers an intrachain picture, with the resulting emission spectrally consistent with that from P3OT, it is sensible to assign this moiety as an ‘Acceptor’ in the usual nomenclature;^77,78^ a fraction of energy initially absorbed by the dye transfers along the chain to P3OT (*cf.* Fig. 4b). However, for the solid bulk format used here it is difficult to rule out contributions from intra-chain energy transfer processes, *i.e.* the dye from one chain being in close enough proximity to the polymer (P3OT) from another. Our focus here has been to demonstrate the proof-of-concept and such discussions and investigations are well beyond the context of the present work. Nevertheless having now established the concept, we feel the selection, placement and control in the positioning of an active species through the “click” functionalization approach does provide an interesting framework from which to explore such questions.

### End-capping of water-soluble polythiophene

**Fig. 5:**
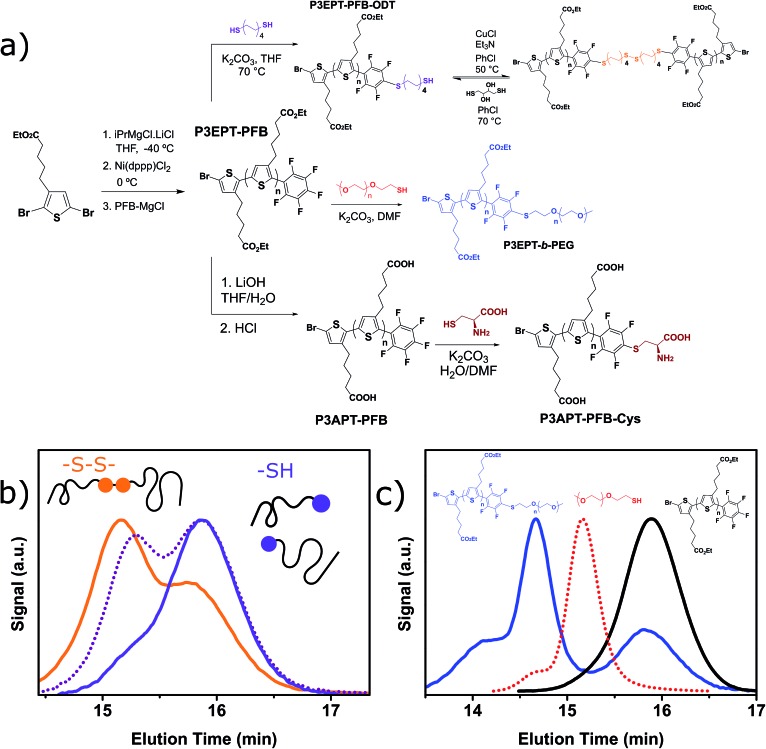
(a) Synthesis of P3EPT–PFB and P3APT–PFB with model “click” reactions performed post-polymerization. (b) Gel permeation chromatography elugram (80 °C in chlorobenzene) of P3EPT–PFB–ODT in the thiol (purple) and disulfide (orange) forms. The dotted purple line illustrates partial reduction of the disulfide link (c) gel permeation chromatography elugram (80 °C in chlorobenzene) of P3EPT–PFB (black), mPEG–SH (red), and P3EPT-*b*-PEG (blue). Traces for all elugrams are obtained from a 450 nm diode array detector, except mPEG–SH which was measured using a refractive index detector.

The interest in water- and/or alcohol-soluble conjugated polymers for applications in biosensing, as well as polyelectrolytes and device interlayers has increased considerably in the recent years.^76–78^ As illustrated in the previous section with 3-MPTS, biotin and Coumarin incorporation, the PFB group can play an essential role in these applications. In an effort to broaden the scope of the PFB-endcapping to water-soluble functionalized polymers, we performed the endcapping procedure on poly(3-(ethylpentanoate)thiophene) (P3EPT), a polymer that can easily be rendered water soluble by ester hydrolysis. P3EPT has previously been synthesized *via* the organozinc-based Rieke method due to the sensitivity of the ester group towards Grignard reagents at temperatures above 0 °C.^79,80^ Utilizing the activating effect of LiCl,^81^ we were able to perform the Grignard metathesis at –40 °C. The excellent solubility of P3EPT in THF allowed us to perform the KCTP at 0 °C, thus reducing the risk of undesired reactions with the ester functionality. The *in situ* endcapping of the P3EPT was performed with 0.1 equivalents of PFB-MgCl at 0 °C for 10 minutes, conditions that were found to be optimal for P3OT (see Fig. 5a).

MALDI-TOF of the resulting polymer (P3EPT–PFB) indicates the formation of >70% mono-PFB endcapped polymer chains (Fig. S12†), analogous to P3OT–PFB. P3EPT–PFB displays a slightly lower molecular weight than P3OT–PFB by GPC (4.3 kg mol^–1^, *Đ* 1.3 *vs.* 5.4 kg mol^–1^, *Đ* 1.3 respectively) which may be attributed to the lower monomer conversion resulting from the lower polymerization temperature. The PFB group exhibits a near identical chemical shift in ^19^F NMR to the P3OT analogue, with the *ortho*, *para* and *meta* fluorine atoms arising at –137.8 ppm, –153.5 ppm and –161.7 ppm.

P3EPT–PFB is highly soluble in polar aprotic solvents such as acetone, ethyl acetate and DMF, opening the door for fast, “click”-like S_N_Ar reactions. Indeed, the reaction of P3EPT–PFB with 1-dodecanethiol (10 eq.) in DMF proceeds to completion in less than 40 minutes at room temperature with K_2_CO_3_ as base (Fig. S21†). This promising result prompted us to investigate the challenging coupling of two polymers to form a block co-polymer. The reduced reactivity of polymer chain ends make this a difficult reaction in polymer science, particularly if one of the polymers has a high number of repeat units.^82^ As such we examined the reaction with 1 equivalent of commercially available *O*-(2-mercaptoethyl)-*O*′-methylpolyethylene glycol (mPEG–SH, *M*_w_ 10 kg mol^–1^) to produce P3EPT-*b*-PEG. After reaction at room temperature for 12 h, the ^19^F NMR (Fig. S24†) demonstrates full substitution of the fluorine at the *para* position of the PFB group and the GPC elugram of P3EPT-*b*-PEG shows a clear shift to higher molecular weight compared to mPEG–SH (Fig. 5c). The elugram also indicates the presence of some remaining P3EPT homopolymer, likely resulting from the *ca.* 30% polymer chains that are not endcapped with PFB and therefore cannot tether to mPEG–SH. This approach offers a transition-metal free alternative to the copper-catalyzed alkyne-azide cycloaddition (CuAAC) that has most commonly been used for the synthesis of P3HT-*b*-PEG.^83^

Thiol functionalized polymers have many potential uses in the context of self-healing polymers,^84,85^ gold and quantum dot surface-modification,^86–89^ and bioconjugation.^90^ P3EPT–PFB was therefore reacted with 1,8-octanedithiol (ODT) in order to generate a thiol-terminated polythiophene. The desired monofunctionalization with ODT was obtained by reacting P3EPT–PFB and ODT (10 eq.) in K_2_CO_3_ and THF for 12 h at 40 °C under argon, thus opening the door for reversible crosslinking and self-healing using disulfide bond formation.^91^ We note that the PFB moiety was found to undergo three SNAr reactions when reacted with an excess of ODT in DMF for 2 hours at room temperature, as evidenced by MALDI-TOF and the two doublets at high chemical shifts in the ^19^F NMR (see ESI†). As a proof of concept, the ODT-functionalized P3EPT–PFB was oxidized to the disulfide using CuCl and triethylamine at 50 °C in chlorobenzene for 12 h. The GPC elugram of the oxidized polymer clearly demonstrates the doubling of molecular weight of a majority of polymer chains (Fig. 5b). As in the case of P3EPT-*b*-PEG, non-PFB endcapped polymer does not change elution time, resulting in a low molecular weight shoulder. Promisingly, the reaction appears to be reversible with treatment with DL-dithiothreitol after 24 h at 70 °C resulting in the reduction of the disulfide link to yield the starting polymer. Note that since the GPC trace of the reduced polymer is identical to the starting material, Fig. 5b shows the trace of the partially reduced polymer, where both species are present.

Finally we were able to prepare the water soluble polymer carboxylate by hydrolysis of P3EPT–PFB with LiOH in THF/H_2_O at room temperature. After acidification, the resulting polymer (P3APT–PFB) could be readily isolated by filtration. Gratifyingly, the PFB endgroup remained intact during this process (Fig. S13†). The P3APT–PFB, which was soluble in PBS buffer, was found to undergo SNAr at the PFB group with the thiol moiety of cysteine in mildly basic aqueous conditions after 7 hours (Fig. S25†). Thus, P3EPT–PFB offers two molecular handles, with the versatility of the PFB end-group demonstrated and the potential for further modifications at the carboxyl sidechain group.

## Conclusion

Through careful optimization of reaction conditions, P3OT was successfully singly end-capped with a PFB group *via* an *in situ* quenching of the KCTP. Temperature was found to significantly influence the distribution of terminal groups of the resulting P3OT. Optimal single end-capping was found to occur after 10 minutes at 0 °C, with a 2 : 1 molar ratio of PFB-MgCl to nickel catalyst.

The resulting polymer was reacted in quantitative yields with a range of nucleophiles including thiols, amines and alcohols under mild conditions. This allowed the proof-of-concept incorporation of three functional moieties. Biotin, a common biomarker used in a variety of biosensing applications, was easily tethered to the P3OT–PFB polymer after thiolation. P3OT–PFB could also be functionalized with 3-MPTS, a commercially available siloxane. Spin coating the resulting polymer on glass substrates followed by annealing afforded a partially retractable film, with up to 20% retention of the P3OT polymer when the glass substrate was treated with piranha solution prior to spin coating. Finally, a thiol-functionalized dye, Coumarin_343_–SH, was incorporated into the polymer through the pseudo “click” substitution of the PFB group. Despite the absorption profiles of the dye and the polymer being only marginally complimentary, signs of non-radiative energy transfer were observed by temporal photoluminescence measurements.

The PFB endcapping procedure was found to be similarly successful on an ester functionalized polythiophene (P3EPT), which was rendered water soluble by ester hydrolysis. In the ester form, P3EPT–PFB exhibited excellent solubility in a range of polar solvents, allowing rapid “click”-type nucleophilic aromatic substitutions at room temperature. The utility of these reactions was demonstrated by forming a diblock co-polymer with a high weight thiolated PEG derivative under transition metal-free conditions. The reaction with excess 1,8-octanedithiol formed mono-thiol containing polymers, which could be reversibly oxidized to higher weight disulfide containing polymers. Finally the water soluble polymer was demonstrated to undergo reaction with the thiol moiety of cysteine under mild conditions, potentially facilitating reaction under biological conditions.

We believe these results demonstrate the utility of the perfluorobenzene end-group as a readily introduced end-capper for conjugated polymers, which can be functionalized under mild conditions by a variety of nucleophiles, affording new opportunities for the development of functional materials.
