# Supplementary material for: Pentafluorobenzene end-group as a versatile handle for para fluoro “click” functionalization of polythiophenes
Source: Chem Sci. 2016 Dec 15;8(3):2215–25. doi: 10.1039/c6sc04427a (PMC5408564; doi:10.1039/c6sc04427a)
Supplement: Supplementary file 1 [file SC-008-C6SC04427A-s001.pdf]

Electronic Supplementary Material (ESI) for Chemical Science.
This journal is © The Royal Society of Chemistry 2016

Pentafluorobenzene end-group as a versatile handle for *para* fluoro "click" functionalization of polythiophenes

*Pierre Boufflet,[‖] Abby Casey,[‖] Yiren Xia,[‡^] Paul N. Stavrinou,[^] Martin Heeney[‖]**

[‖]Department of Chemistry and Centre for Plastic Electronics, Imperial College London, Exhibition Rd, London SW7 2AZ, U.K.

[‡]Department of Physics and Centre for Plastic Electronics, Imperial College London, Exhibition Rd, London SW7 2AZ, U.K.

^Department of Engineering Science, University of Oxford, Parks Road, Oxford OX1 3PJ, U.K.

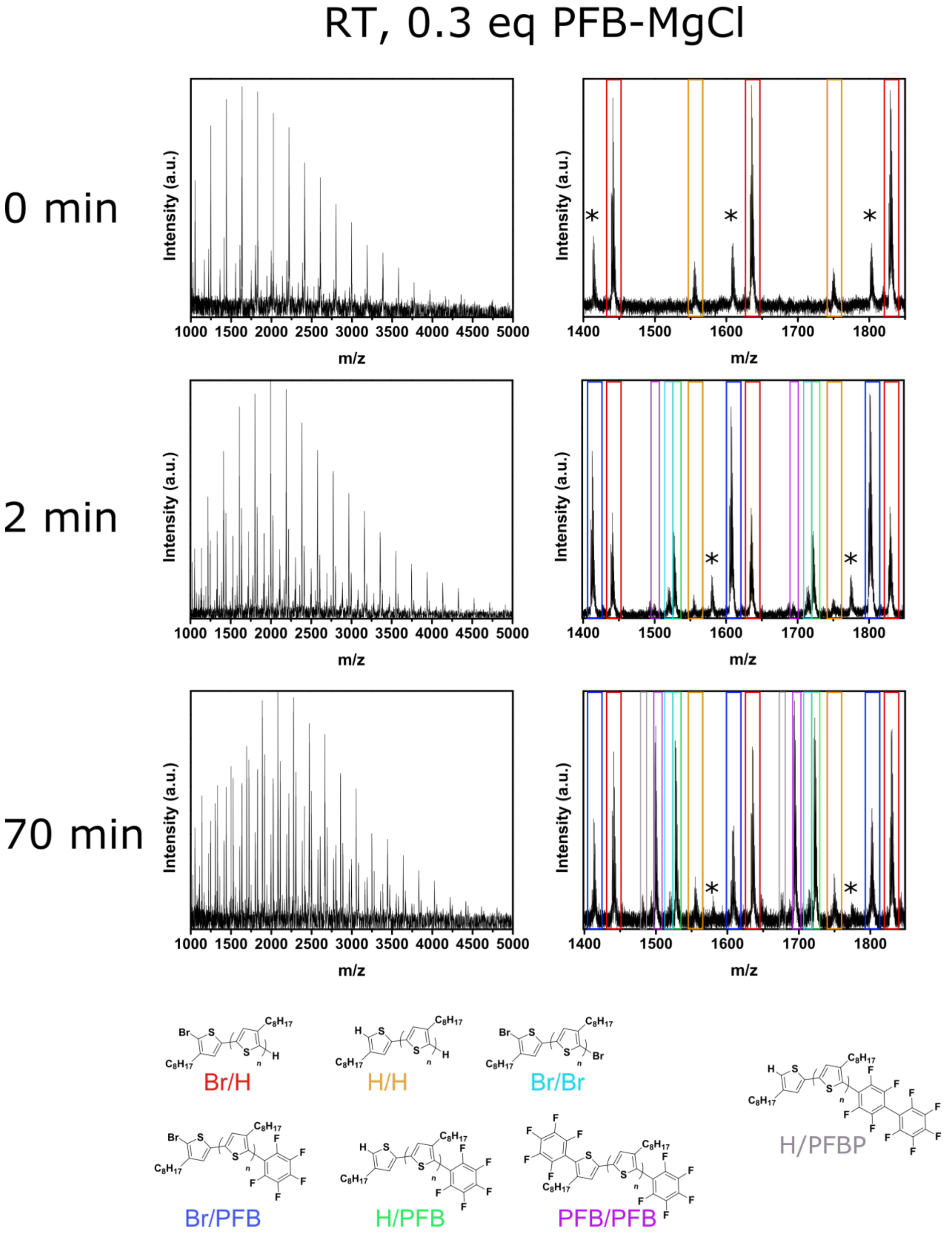

Figure S1. Wide (left) and expanded (right) matrix assisted laser desorption-ionization time-of-flight measurements (MALDI-TOF) of aliquots from the GRIM polymerization of 2,5-dibromo-3-octylthiophene illustrating the evolution of end-groups as a function of time after adding 0.3 equivalents of PFB-MgCl (relative to 2,5-dibromo-3-octylthiophene) at room temperature. All samples were quenched with 5M HCl before extraction with $CHCl_3$ for analysis. 0 min is an aliquot taken prior to the addition of PFB-MgCl. Signals marked with an asterisk (*) could not be attributed to a reasonable end-group combination, particularly as they are present prior to PFB-MgCl addition. Since they tend to track the most abundant signal (difference of -26) and are not present in analogous experiments with end-capping experiments of 2,5-dibromo-3-hexylthiophene, these are tentatively attributed to fragmentation of the 3-octylthiophene repeat unit.

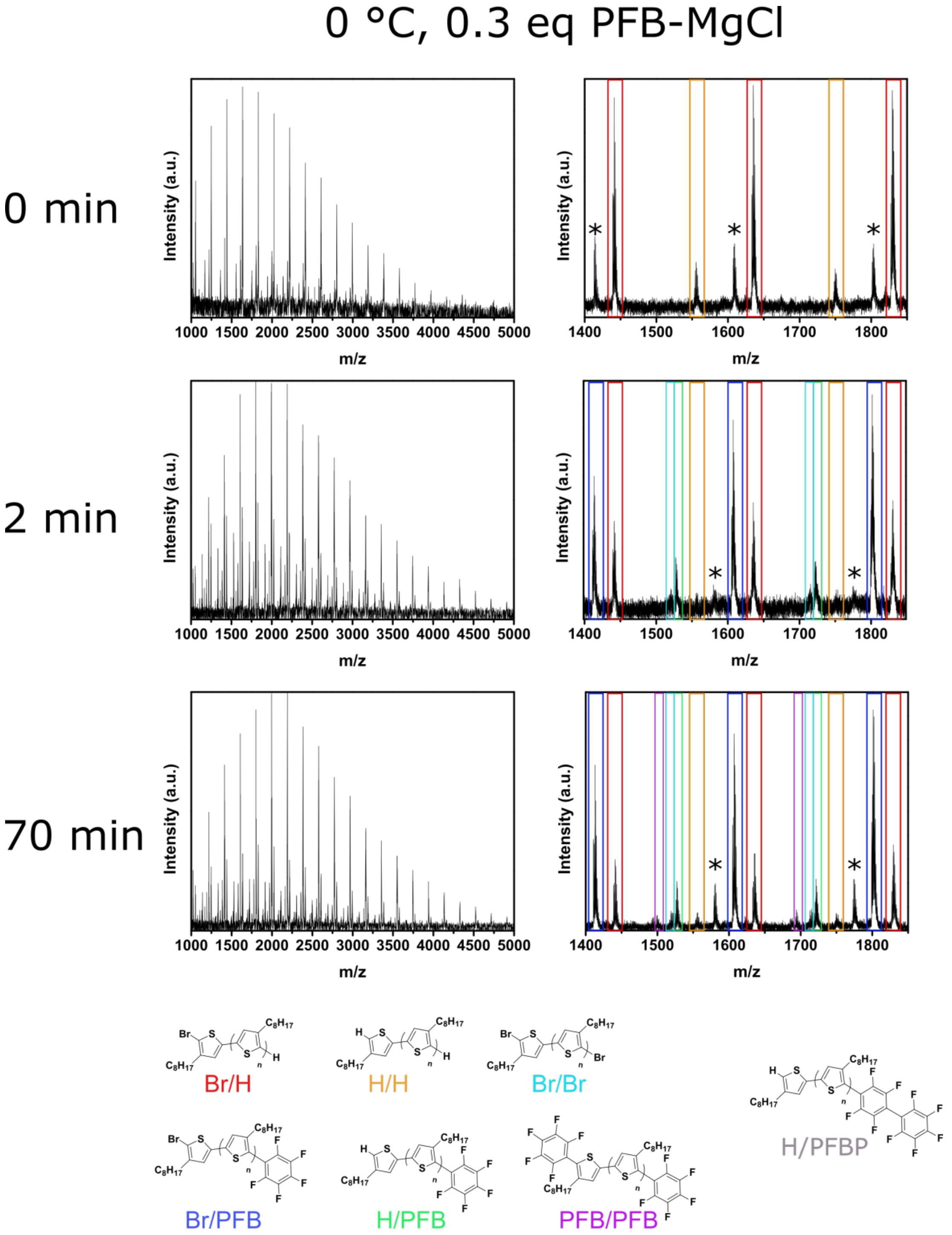

Figure S2. Wide (left) and expanded (right) matrix assisted laser desorption-ionization time-of-flight (MALDI-TOF) measurements of aliquots from the GRIM polymerization of 2,5-dibromo-3-octylthiophene illustrating the evolution of end-groups as a function of time after adding 0.3 equivalents of PFB-MgCl (relative to 2,5-dibromo-3-octylthiophene) at 0 °C. All samples were quenched with 5M HCl before extraction with $CHCl_3$ for analysis. 0 min is an aliquot taken prior to the addition of PFB-MgCl. Signals marked with an asterisk (*) could not be attributed to a reasonable end-group combination, particularly as they are present prior to PFB-MgCl addition. Since they tend to track the most abundant signal (difference of -26) and are not present in analogous experiments with end-capping experiments of 2,5-dibromo-3-hexylthiophene, these are tentatively attributed to fragmentation of the 3-octylthiophene repeat unit.

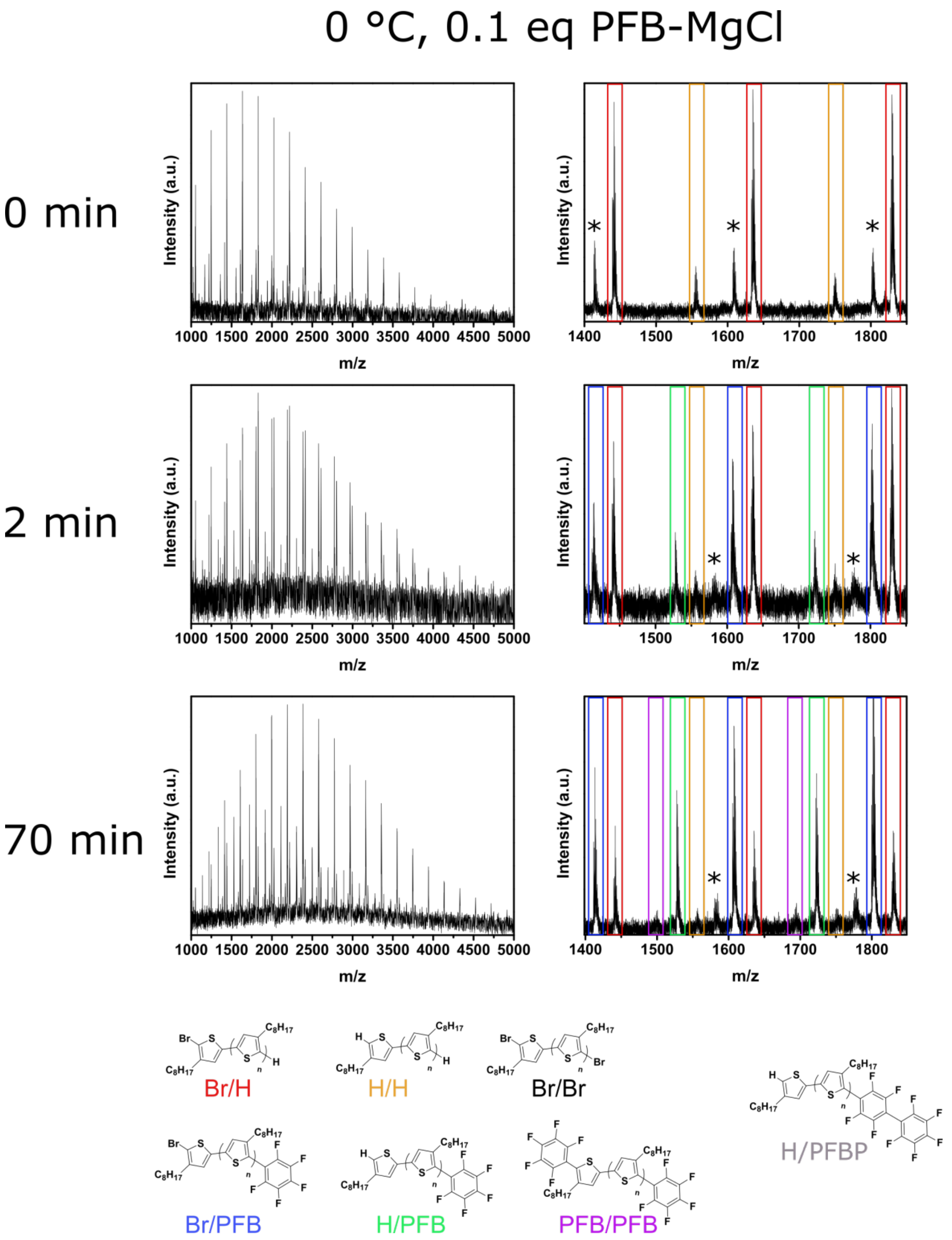

Figure S3. Wide (left) and expanded (right) matrix assisted laser desorption-ionization time-of-flight (MALDI-TOF) measurements of aliquots from the GRIM polymerization of 2,5-dibromo-3-octylthiophene illustrating the evolution of end-groups as a function of time after adding 0.1 equivalents of PFB-MgCl (relative to 2,5-dibromo-3-octylthiophene) at 0 °C. All samples were quenched with 5M HCl before extraction with $CHCl_3$ for analysis. 0 min is an aliquot taken prior to the addition of PFB-MgCl. Signals marked with an asterisk (*) could not be attributed to a reasonable end-group combination, particularly as they are present prior to PFB-MgCl addition. Since they tend to track the most abundant signal (difference of -26) and are not present in analogous experiments with end-capping experiments of 2,5-dibromo-3-hexylthiophene, these are tentatively attributed to fragmentation of the 3-octylthiophene repeat unit.

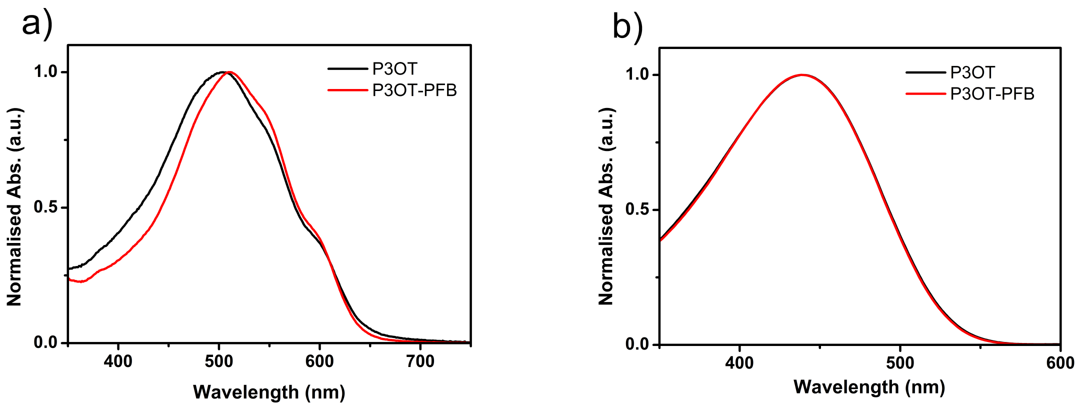

Figure S4. Normalised UV-visible spectra (a) thin film (spun from chloroform) and (b) solution (chloroform) of P3OT and P3OT-PFB.

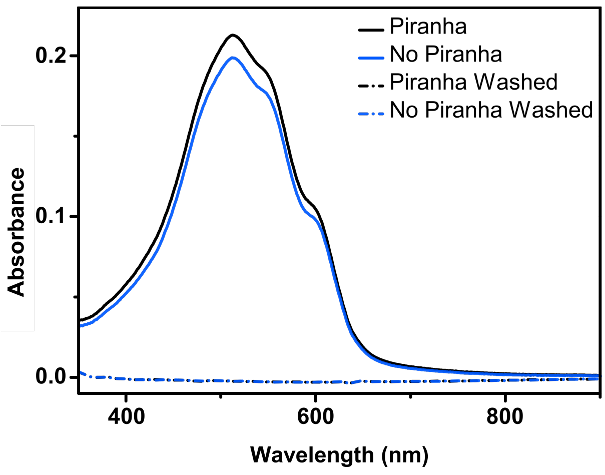

Figure S5. Control experiment of UV-visible spectrum for thin-film retention of unmodified P3OT-PFB. Films spun from 5 mg/mL solution in THF.

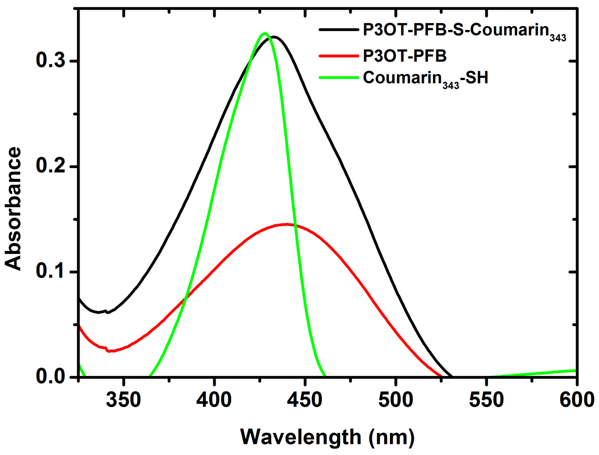

Figure S6. Solution UV-visible spectra of P3OT-PFB (16 µg/mL in THF), P3OT-PFB-S-Coumarin343 (16 µg/mL in THF), and thiolated Coumarin$_{343}$ (7.8 µg/mL in THF).

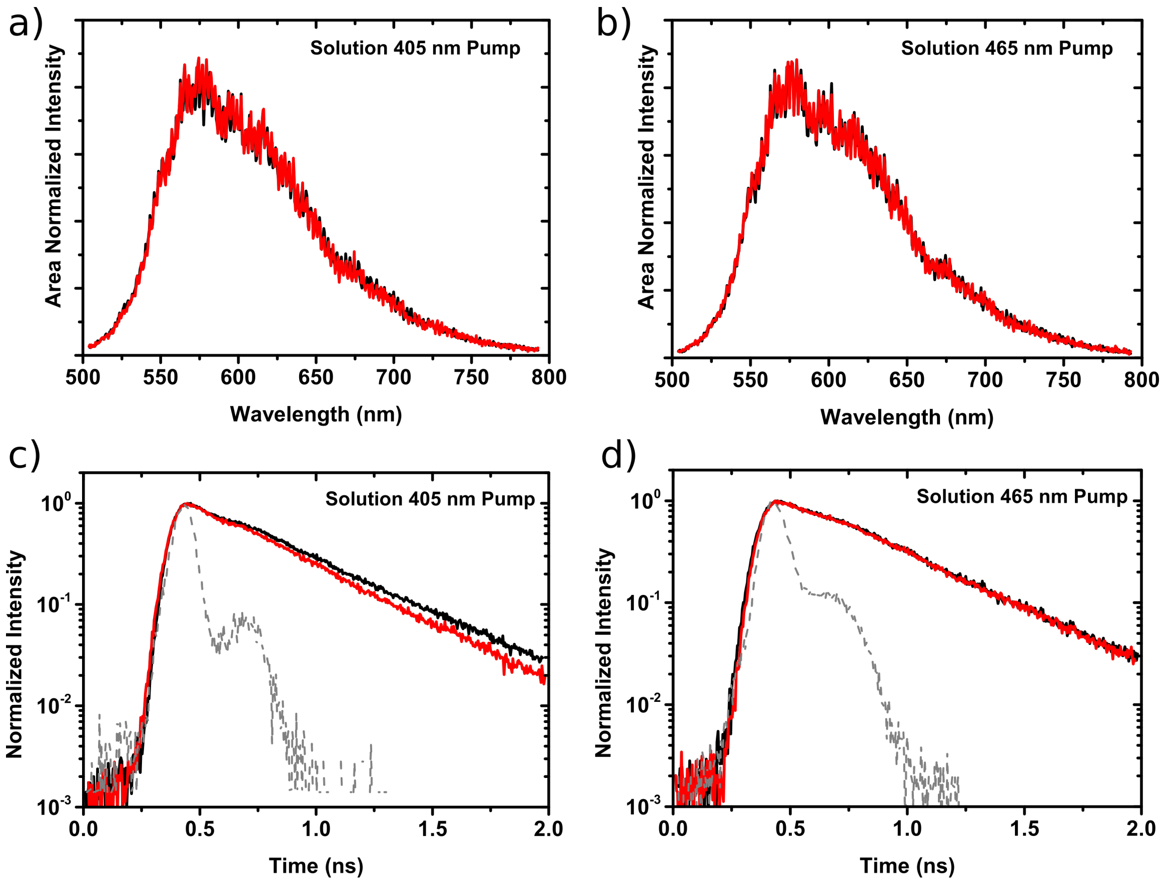

Figure S7. Photoluminescence spectra, taken from the streak camera, for solutions of P3OT-PFB (red) and P3OT-PFB-Coumarin$_{343}$ (black) at 405 nm (a) and 465 nm (b) excitation wavelengths. Transient photoluminescence, over the full wavelength range in (a) and (b) for P3OT-PFB (red) and P3OT-PFB-Coumarin$_{343}$ (black) at 405 nm (a) and 465 nm (b) excitation wavelengths. The response from the excitation source is also shown.

Table S7-1. Summary of extracted lifetimes for samples under study. THF Solutions of P3OT-PFB and P3OT-PFB-Coumarin$_{343}$.

Excitation wavelength	P3OT-PFB-Coumarin$_{343}$	P3OT-PFB
405 nm	0.40 ns	0.36 ns
465 nm	0.37 ns	0.37 ns

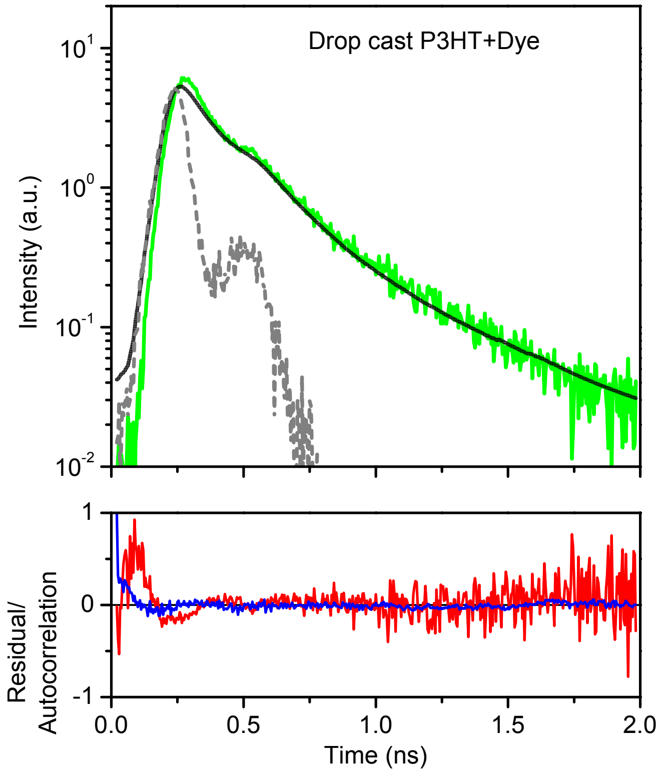

Figure S8. An example of the laser convolved with single exponential fitting procedure for extracting lifetimes for a drop cast sample of P3HT+Dye lifetime. Upper plot shows the recorded sample response (green), the excitation laser response at 405 nm (grey dotted) and the resulting convolved fit (black). Lower panel displays the residual (blue) and autocorrelation traces (red).

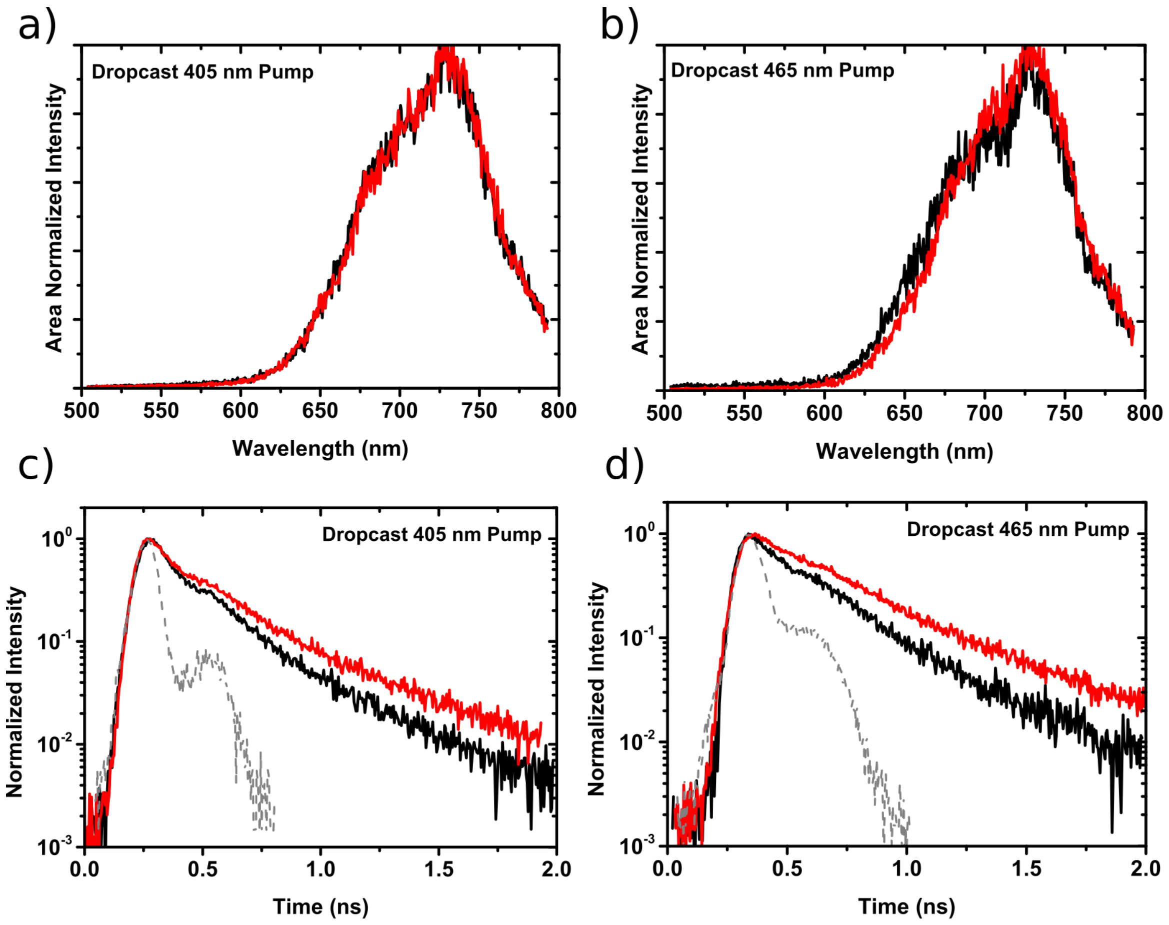

Figure S9. Photoluminescence spectra, taken from the streak camera, of P3OT-PFB (red) and P3OT-PFB-Coumarin$_{343}$ (black) dropcast samples at 405 nm (a) and 465 nm (b) pump wavelength. Temporal photoluminescence of P3OT-PFB (red) and P3OT-PFB-Coumarin$_{343}$ (black) at 405 nm (a) and 465 nm (b) excitation wavelengths. The response from the excitation source is also shown.

Table S9-1. Summary of extracted lifetimes for samples under study. Drop cast samples of P3OT-PFB and P3OT-PFB-Coumarin$_{343}$.

Excitation wavelength	P3OT-PFB-Coumarin$_{343}$	P3OT-PFB
405 nm	0.22 ns	0.27 ns
465 nm	0.27 ns	0.37 ns

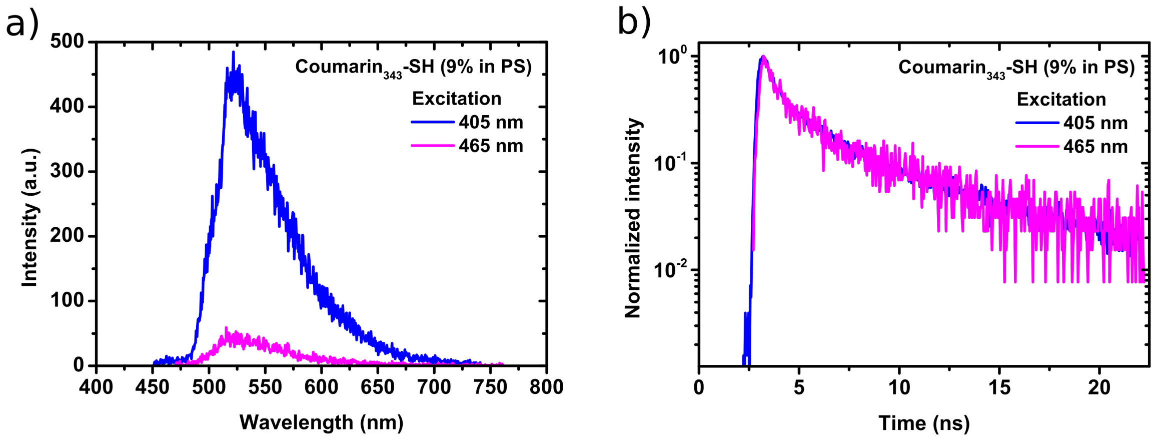

Figure S10. Photoluminescence spectra (a), taken from the streak camera, and transient photoluminescence (b) measurements of 9 wt.% $Coumarin_{343}$-SH in polystyrene, spin cast from THF solutions. The spectra displayed for two exciation wavelengths, 405 nm and 465 nm. The extracted lifetimes (expectation values) for both excitation wavelengths are found to be around 4 ns.

Experimental

Reagents and chemicals were purchased from commercial sources such as Sigma-Aldrich, Acros, Rieke Metals etc. unless otherwise stated. All reactions were carried out using solvents and reagents as commercially supplied, unless otherwise stated. Ethyl 5-(2,5-dibromothiophen-3-yl)pentanoate[1] and thiolated biotin derivative (Biotin-SH)[2] were synthesised as previously reported. Titrations of Grignard reagents were performed according to the prcedure outlined by Krasovskiy & Knochel.[3] ^{1}H, ^{19}F, and ^{13}C NMR spectra were recorded on a Bruker AV-400 (400 MHz) instrument, using the residual solvent resonance of $CDCl_3$ or DMSO-d^6 as reference, and values are indicated in ppm.

MALDI-TOF measurements were performed with a Waters MALDI Micro MX spectrometer in reflection mode on a stainless steel plate. 1 µL of a solution of terthiophene (or trans-2-[3-(4-t-butyl-phenyl)-2-methyl-2-propenylidene]malononitrile (DCTB) for P3EPT polymers) dissolved in chloroform (20 mg/mL) was deposited on the sample target area, and once dry, 1 µL of a solution of the sample in chloroform (0.016 mg/mL) was deposited on the same target area. Once dry, the sample was immediately measured using ca. 75% laser intensity, and the target area was scanned to find >5 spectra with an optimal signal-to-noise ratio. The resulting spectra obtained were averaged using the built in functions from the Masslynx software. Each spectrum was then normalised against the largest peak signal attributed to a polymer/endcapper combination. The intensities of each peak attributed to all termination possibilities of the most abundant repeat unit were then extracted from the spectra to obtain the approximate relative end-capping composition. Samples for MALDI-TOF experiments used for end-capping optimisation were obtained by taking 0.1 mL aliquots of the

reaction mixture and quenching them in 5M HCl, followed by filtering and washing the resulting polymer with methanol.

Number-average (Mn) and Weight-average (Mw) were determined by Agilent Technologies 1200 series GPC running in chlorobenzene at 80°C, using two PL mixed B columns in series, and calibrated against narrow polydispersity polystyrene standards.

UV-vis spectra were recorded on a Shimadzu UV-1800 spectrometer, with solution UV-vis measured in chloroform at a concentration of 0.016 mg/mL, and thin films were spin coated on fused silica slides at 2000 rpm from a 5 mg/mL solution. Prior to spin coating, glass slides were washed by successive sonication for 10 minutes in a 10:1 water/Decon 90 mixture, distilled water, acetone, and isopropanol, before being stored in fresh isopropanol. Slides to be treated with Piranha solution for the surface modification were then dried in an oven at 140 ºC for 10 minutes, before being immersed in a freshly prepared Piranha solution (4:1 conc. H_2SO_4/H_2O_2 (30% aq.)) for 30 minutes. The slides were then used immediately after being rinsed with distilled water.

The transient photoluminescence characteristics are measured under ambient conditions at room temperature using a streak camera. Samples are excited by a picosecond light pulser controlled diode laser (Hamamatsu PLP-10). The emission is guided into a monochromator (Chromex 250is) and finally measured by streak scope (Hamamatsu C4334-01). Excitation and emission are synchronized by a delay generator (Hamamatsu C4792-01) controlled and calibrated via PC software. In a single measurement, signal is displayed in matrix form 640 channels (wavelength) by 480 channels (time), which records 290 nm wavelength range and a user-defined acquisition time range (from 1 ns to 1 ms). The setup has a 6 nm spectral resolution, while temporal resolution depends on the acquisition time range. The minimum laser FWHM obtained is 0.1 ns in 1 ns time range. The laser pulse frequency is fixed at 1MHz, and the streak scope always operates at 10Hz for photon counting. We take the sum of the counts of thousands of exposures (pulses) to achieve a reliable statistical average.

Synthesis of Coumarin$_{343}$-SH:

Ethylcarbodiimide.hydrochloride (0.403 g, 2.11 mmol) was added to a solution of coumarin 343 (0.500 g, 1.75 mmol) and N-hydroxysuccinimide (NHS) (0.302 g, 2.63 mmol) in DCM (50 mL) and stirred at room temperature for 24 h. The solvent was evaporated and the solid washed with EtOH/AcOH/water (95:1:4 v:v:v). (Intermediate product confirmed by ^{1}H NMR (400 MHz, CDCl3) δ (ppm): 8.46 (s, 1H), 6.95 (s, 1H), 3.42 – 3.34 (m, 4H), 2.92 – 2.84 (m, 6H), 2.80 – 2.72 (m, 2H), 2.03 – 1.92 (m, 4H).) After drying, the NHS-ester of coumarin (0.603 g, 1.58 mmol) was added to a mixture of cysteamine hydrochloride (0.179, 1.58 mmol) and *N*-methylmorpholine (NMM) (0.351 g, 3.47 mmol) in DMF/water (6:1, v:v, 40 mL). The reaction mixture was stirred for 24 h before DL-dithiothreitol (0.487 g, 3.15 mmol) was added and the reaction stirred for a further 20 h. The reaction mixture was then diluted with DCM (100 mL) and washed with water (3 x 50 mL). The solvent was then removed *in vacuo* and the crude product purified by column chromatography using ethyl acetate/hexane (8:2, v:v) as eluent to afford Coumarin$_{343}$-SH as a yellow solid (0.200 g, 33% over two steps). ^{1}H NMR (400 MHz, $CDCl_3$) δ (ppm): 9.14 (br t, *J* = 5.8 Hz, 1H), 8.59 (s, 1H), 7.01 (s, 1H), 3.68 – 3.59 (m, 2H), 3.37 – 3.28 (m, 4H), 2.92 – 2.84 (m, 2H), 2.81 – 2.71 (m, 4H), 2.03 – 1.92 (m, 4H), 1.46 (t, *J* = 8.5 Hz, 1H). ^{13}C NMR (101 MHz, CDCl3) δ (ppm): 163.76, 163.03, 152.68, 148.23, 148.14, 127.03, 119.67, 108.63, 108.20, 105.63, 50.24, 4.9.82, 42.80, 27.45, 24.54, 21.11, 20.17, 20.08. MS(ES+): m/z = 345.1 [M+H]

Typical procedure for the synthesis of P3OT-PFB:

In a dry 20 mL vial with a stirrer bar, 2,5-dibromo-3-octylthiophene (600 mg, 1.69 mmol) was dissolved in dry THF (15.6 mL) under argon, and to the stirred solution was added freshly titrated isopropylmagnesium chloride lithium chloride complex solution (1.37 mL, 1.2 M solution in THF) dropwise. After 1 h, dichloro[1,3-bis(diphenylphosphino)propane]nickel (46 mg, 0.085 mmol) was added in one portion, and the resulting crimson polymerisation solution was stirred at room temperature for 1 h.

Meanwhile, in a dry 2-5 mL vial, Iodopentafluorobenzene (117 mg, 0.40 mmol) was dissolved in dry THF (0.6 mL) under argon, and cooled to -78 °C. To the stirred solution was then added butylmagnesium chloride (0.20 mL, 2 M in THF), and the solution stirred for 15 minutes, then maintained at -78 °C until its use in the next step.

The polymerisation solution was then cooled to 0 °C in an ice bath before adding freshly prepared pentafluorophenylmagnesium chloride (0.34 mL, 0.5 M solution in THF) dropwise. After further stirring for 10 minutes at 0 °C, the reaction was quenched with aqueous HCl (1.00 mL, 5 M), stirred for 2 minutes, and precipitated into methanol. The precipitate was filtered through a Soxhlet thimble, and purified by Soxhlet extraction, washing successively with methanol and acetone, and finally extracting with chloroform. The chloroform was removed *in vacuo*, and the resulting polymer reprecipitated from hot chloroform into methanol, filtered, and dried under vacuum (180 mg, 54%). ^{1}H NMR (400 MHz, $CDCl_3$) δ (ppm): 6.98 (br s, 1H), 2.94 – 2.51 (m, 2H), 1.80 – 1.61 (m, 2H), 1.49 – 1.18 (m, 10H), 0.97 – 0.82 (m, 3H). ^{19}F NMR (377 MHz, $CDCl_3$) δ (ppm): -137.83 (dd, *J* = 23, 8 Hz), -153.49 (t, *J* = 21 Hz), -161.30 – -162.29 (m).

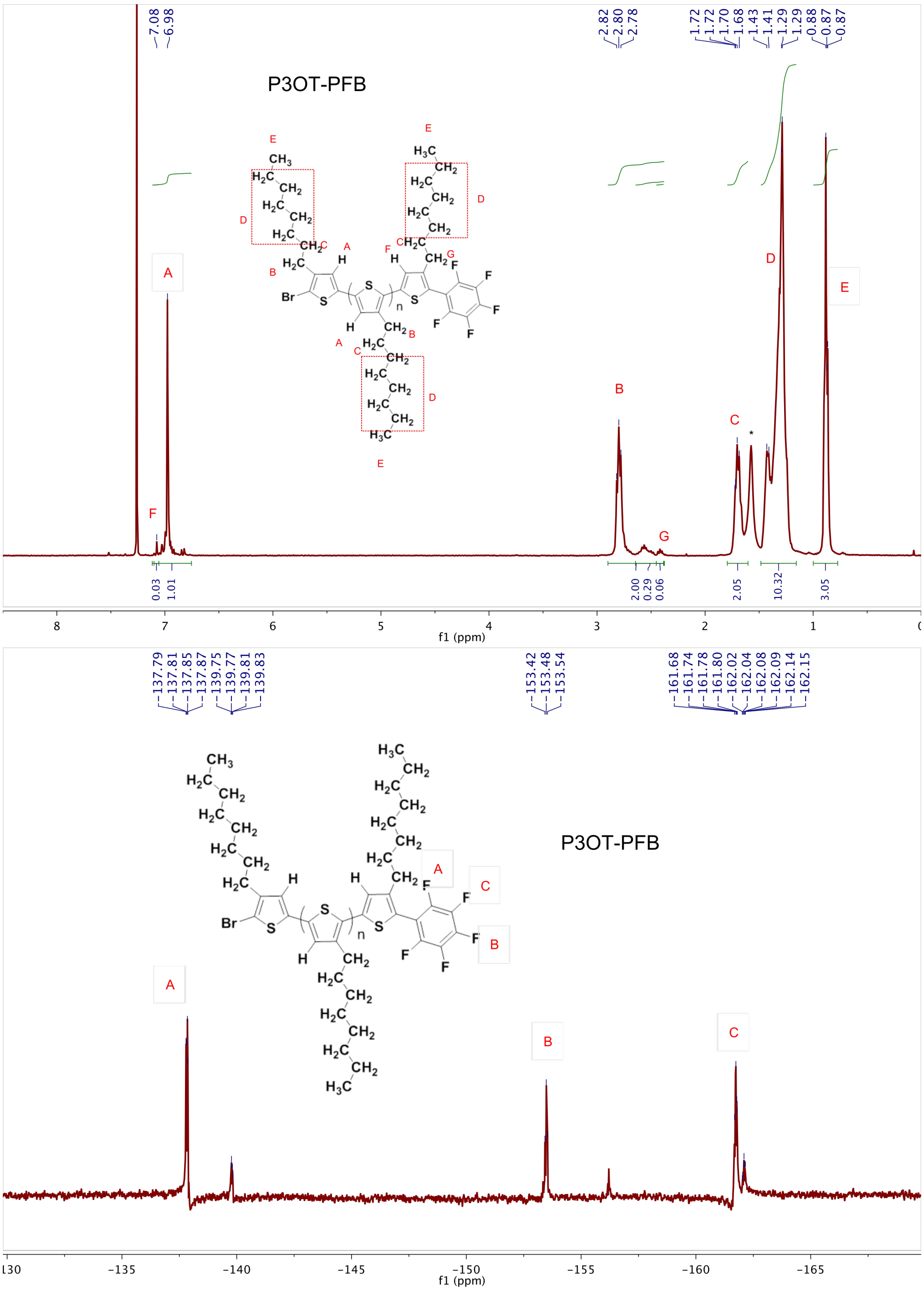

Figure S11. ^{1}H (top) and ^{19}F (bottom) NMR or P3OT-PFB in $CDCl_3$

Synthesis of PFB-terminated poly-2,5-diyl-(3-ethylpentanoate)thiophene (P3EPT-PFB):

In a dry 20 mL vial with a stirrer bar, ethyl 5-(2,5-dibromothiophen-3-yl)pentanoate (703 mg, 1.90 mmol) was dissolved in dry THF (17 mL) under argon, and cooled to -40 °C. To the stirred solution was added freshly titrated isopropylmagnesium chloride lithium chloride complex solution (1.53 mL, 1.2 M solution in THF) dropwise. After 1 h, the reaction mixture was warmed to 0 °C, and dichloro[1,3-bis(diphenylphosphino)propane]nickel (51 mg, 0.95 mmol) was added in one portion. The resulting orange polymerisation solution was stirred at 0 °C for 1 h.

Meanwhile, in a dry 2-5 mL vial, Iodopentafluorobenzene (117 mg, 0.40 mmol) was dissolved in dry THF (0.6 mL) under argon, and cooled to -78 °C. To the stirred solution was then added butylmagnesium chloride (0.20 mL, 2 M in THF), and the solution stirred for 15 minutes, then maintained at -78 °C until its use in the next step.

Freshly prepared pentafluorophenylmagnesium chloride (0.34 mL, 0.5 M solution in THF) was then added dropwise to the polymerisation mixture at 0°C. After further stirring for 10 minutes at 0 °C, the reaction was quenched with aqueous HCl (1.00 mL, 5 M), stirred for 2 minutes, and precipitated into methanol. The precipitate was filtered through a Soxhlet thimble, and purified by Soxhlet extraction, washing successively with methanol and hexane, and finally extracting with THF. The THF was removed *in vacuo*, and the resulting polymer reprecipitated from hot chloroform into cold methanol, filtered, and dried under vacuum (186 mg, 47%). ^{1}H NMR (400 MHz, $CDCl_3$) δ (ppm): 6.98 (s, 1H), 4.13 (q, *J* = 7.1 Hz, 2H), 2.96 – 2.51 (m, 2H), 2.43 – 2.31 (m, 2H), 1.83 – 1.68 (m, 4H), 1.25 (t, *J* = 7.0 Hz, 3H). ^{19}F NMR (377 MHz, $CDCl_3$) δ (ppm): -137.80 (dd, *J* = 23, 7 Hz), -153.11 (t, *J* = 21 Hz), -161.40 – -161.53 (m).

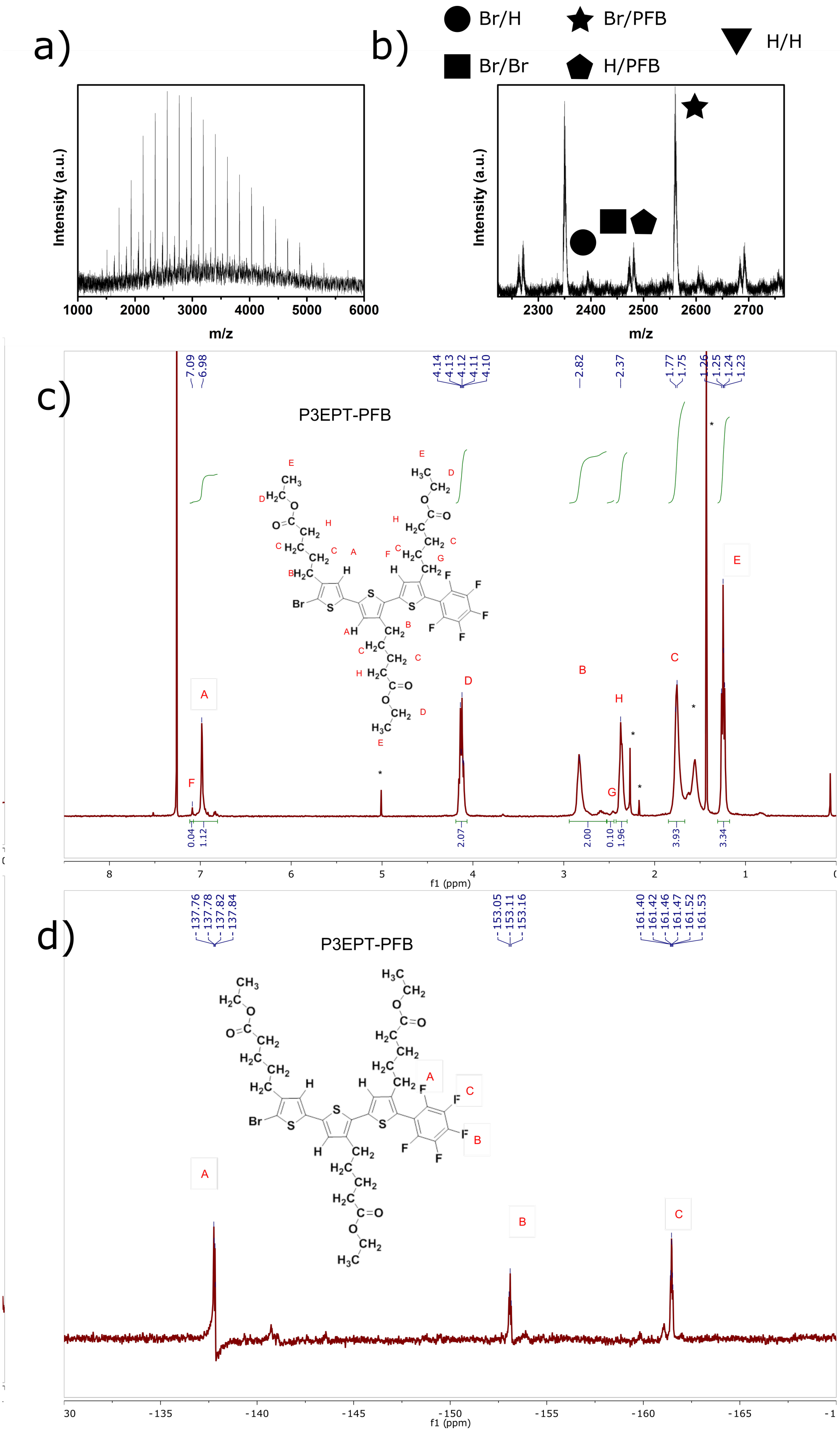

Figure S12. a) MALDI-TOF spectrum of P3OT-PFB. b) Selected region of MALDI-TOF spectrum with signals assigned. c) and d) are respectively ^{1}H and ^{19}F NMR of P3OT-PFB in $CDCl_3$. Starred signals are assigned to residual solvent.

Synthesis of PFB-terminated poly-2,5-diyl-(3-pentanoic acid)thiophene (P3APT-PFB):

P3EPT-PFB (40 mg, 0.19 mmol relative to monomer) was dissolved in THF (1 mL) and water (1 mL). LiOH (10 mg, 42 mmol) was added, and the reaction stirred at room temperature for 12 hours. The reaction mixture was then poured into water (20 mL), and the aqueous phase was washed with diethyl ether (10 mL) and hexane (10 mL). The aqueous phase was then acidified with 5M HCl, and filtered. The filtrate was washed with acetonitrile, and dried under vacuum (26 mg, 85%). ^{1}H NMR (400 MHz, DMSO) δ (ppm): 12.01 (s, 1H), 7.37 – 7.04 (m, 1H), 2.89 – 2.71 (m, 2H), 2.33 – 2.20 (m, 2H), 1.68 – 1.51 (m, 4H). ^{19}F NMR (377 MHz, DMSO) δ (ppm): -139.01 (dd, *J* = 24, 6 Hz), -153.63 (t, *J* = 23 Hz), -161.70 – -162.39 (m).

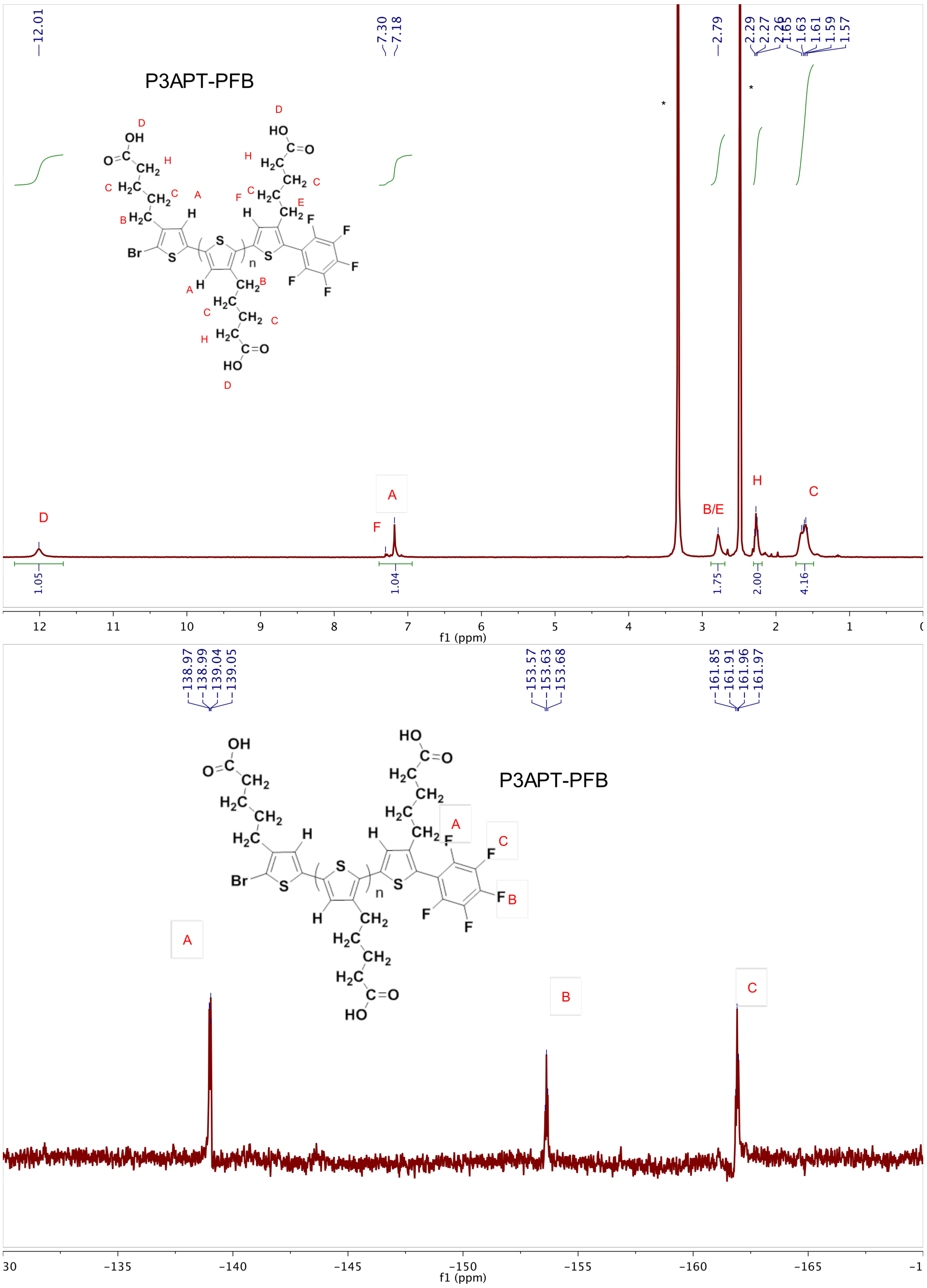

Figure S13. ^{1}H (top) and ^{19}F (bottom) NMR or P3APT-PFB in DMSO-d^{6}

Typical procedure for nucleophilic aromatic displacement on P3OT-PFB (Typical S_NAr procedure):

Conditions A - Base: K_2CO_3, Solvent: THF

Conditions B - Base: K_2CO_3, Solvent: THF/DMF 4:1

Conditions C - Base: KOH, Solvent: THF

Conditions D – Base: K_2CO_3, Solvent: DMF

In a 0.5 – 2 mL pressure-resistant vial with a stirrer bar, P3OT-PFB or P3EPT-PFB (0.010 mmol (eg. 20 mg when Mn from MALDI-TOF is approx. 2000 g mol^{-1})), and solvent (0.5 mL) were added, and the solution gently heated and stirred until all the polymer had visibly dissolved, after which the nucleophile (10 eq., 0.1 mmol) and base (15 eq, 0.150 mmol) were added. The vial was then sealed, and stirred at the indicated temperature. A sample was taken from the reaction mixture at given intervals and analyzed by MALDI-TOF to follow the consumption of PFB-containing polymer chains. Once all PFB-end groups were consumed, the reaction was poured into water (5 mL), then methanol (5 mL) was added and the precipitate was filtered. The solid was washed with acetone (for P3OT-PFB) or hexane (for P3EPT-PFB) to remove excess nucleophile. For less soluble nucleophiles (Biotin-SH and $Coumarin_{343}$-SH), the resulting precipitate was washed with methanol and acetone by Soxhlet extraction.

Synthesis of P3OT-PFB-$SC_{12}H_{25}$:

Prepared according to conditions A of typical S_NAr procedure using dodecanethiol at 70 °C for 12 hours.

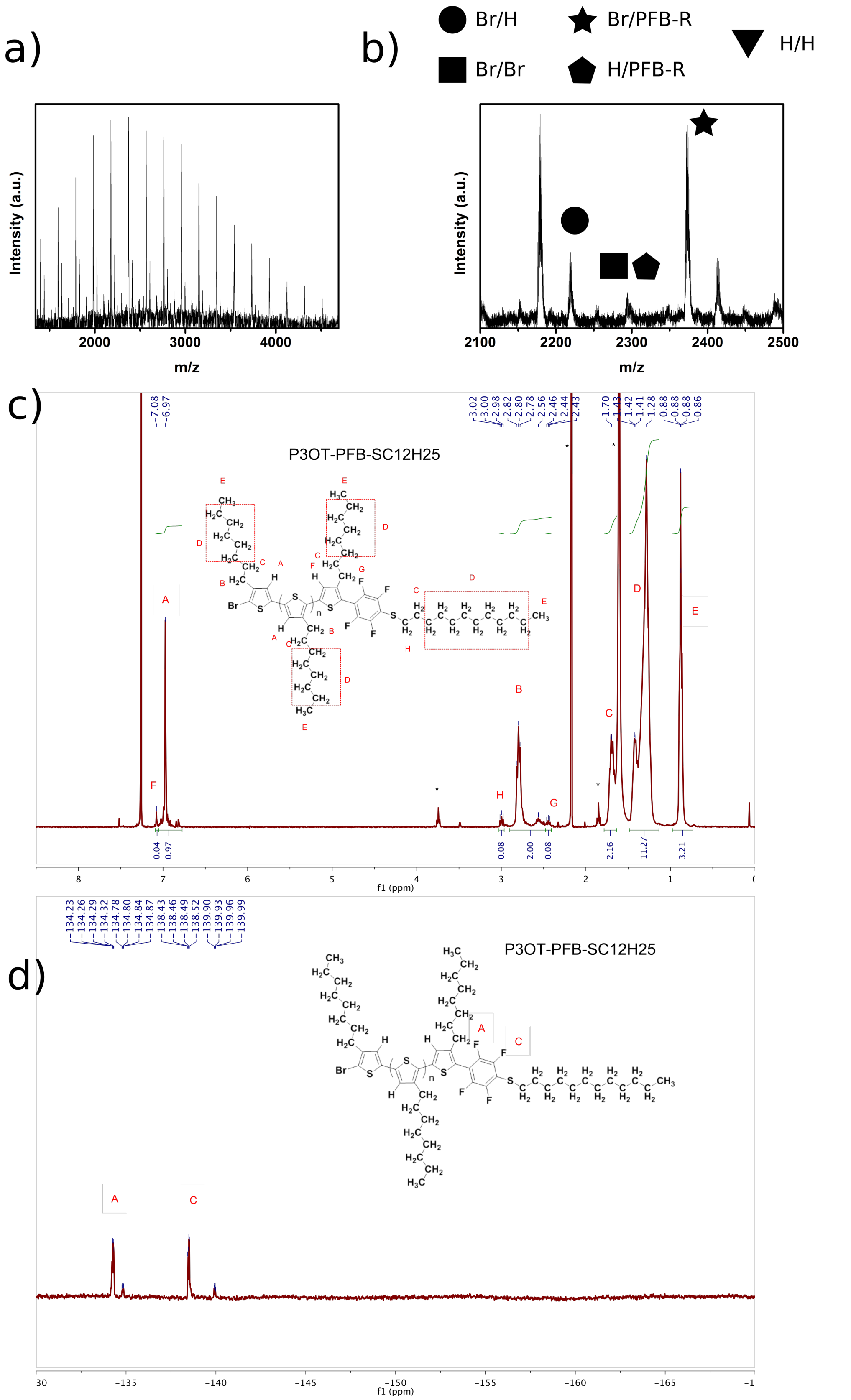

Figure S14. a) MALDI-TOF spectrum of P3OT-PFB-$SC_{12}H_{25}$. b) Selected region of MALDI-TOF spectrum with signals assigned, where R = -$SC_{12}H_{25}$. c) and d) are respectively ^{1}H and ^{19}F NMR of P3OT-PFB-$SC_{12}H_{25}$ in $CDCl_3$. Starred signals are assigned to residual solvent.

Synthesis of P3OT-PFB-STol:

Prepared according to conditions A of typical S_NAr procedure using 4-mercaptotoluene at 70 °C for 12 hours.

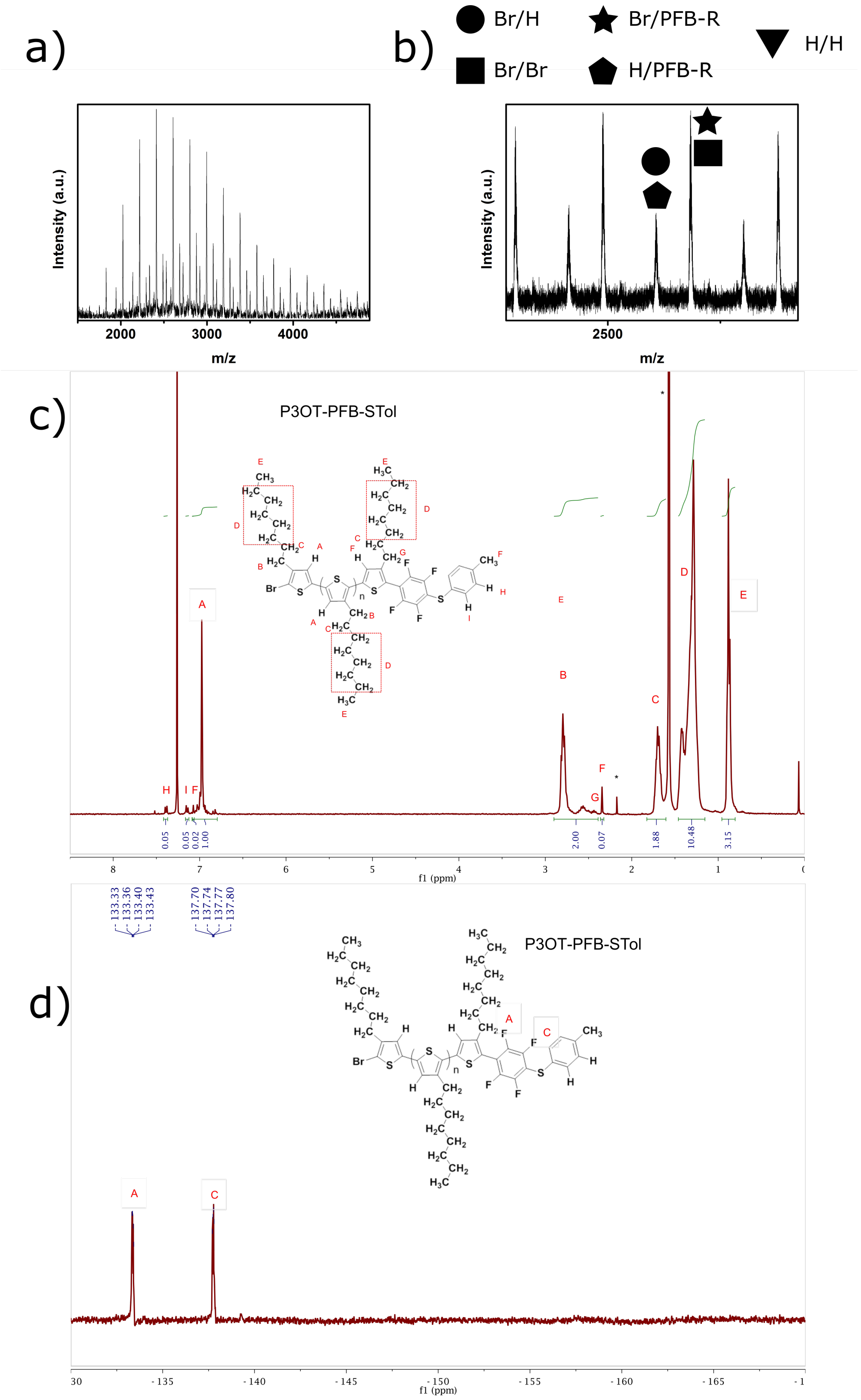

Figure S15. a) MALDI-TOF spectrum of P3OT-PFB-STol. b) Selected region of MALDI-TOF spectrum with signals assigned, where R = -STol. c) and d) are respectively ^{1}H and ^{19}F NMR of P3OT-PFB-STol in $CDCl_3$. Starred signals are assigned to residual solvent.

Synthesis of P3OT-PFB-NC_8H_{17}:

Prepared according to conditions B of typical S_NAr procedure using octylamine at 70 °C for 12 hours.

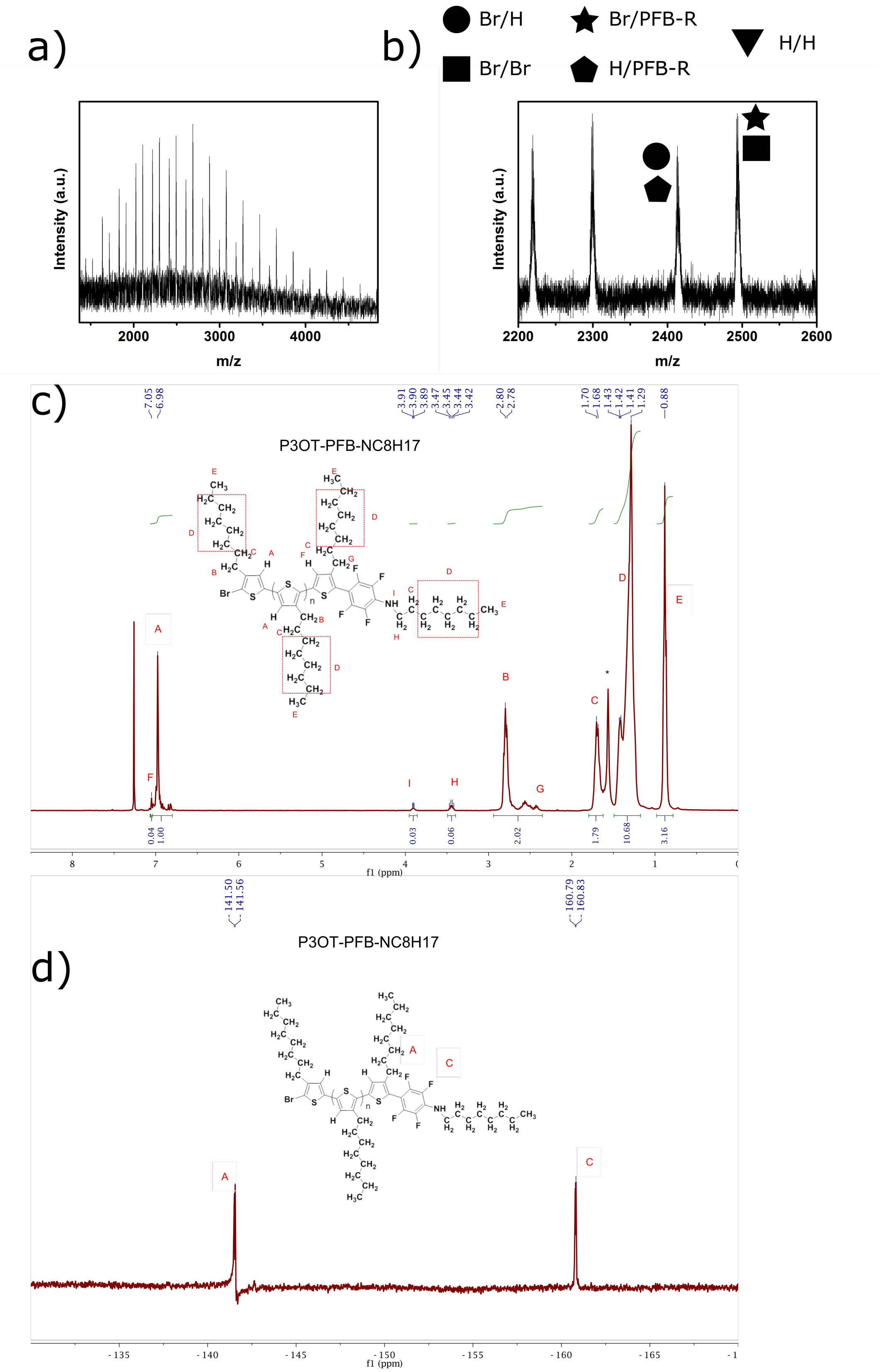

Figure S16. a) MALDI-TOF spectrum of P3OT-PFB-NC_8H_{17}. b) Selected region of MALDI-TOF spectrum with signals assigned, where R = -NC_8H_{17}. c) and d) are respectively ^1H and ^{19}F NMR of P3OT-PFB-NC_8H_{17} in $CDCl_3$. Starred signals are assigned to residual solvent.

Synthesis of P3OT-PFB-(OC_5H_{11})$_2$:

Prepared according to conditions C of typical S_NAr procedure using pentanol at 70 °C for 12 hours.

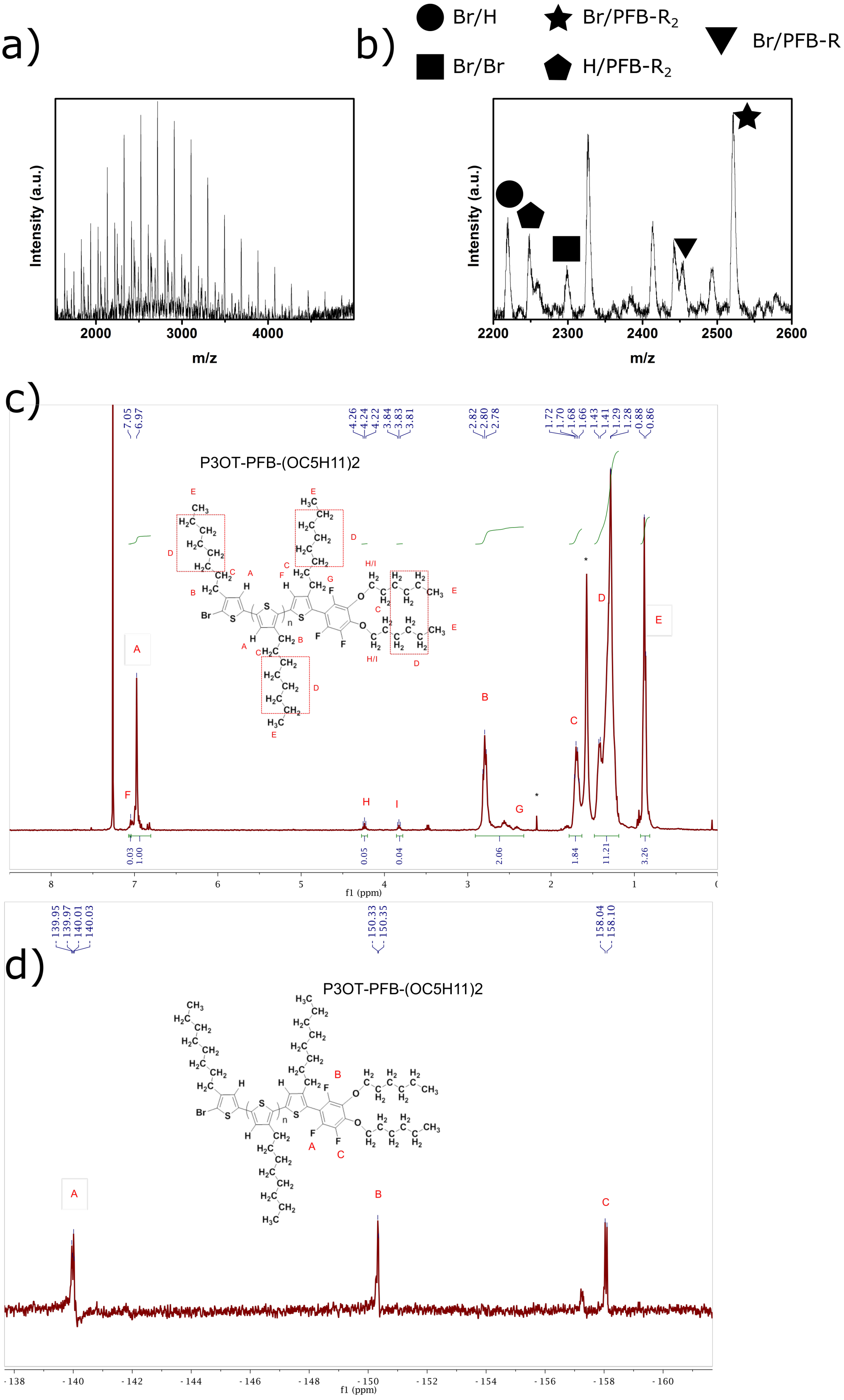

Figure S17. a) MALDI-TOF spectrum of P3OT-PFB-$(OC_5H_{11})_2$. b) Selected region of MALDI-TOF spectrum with signals assigned, where R = $-OC_5H_{11}$. c) and d) are respectively ^{1}H and ^{19}F NMR of P3OT-PFB-$(OC_5H_{11})_{27}$ in $CDCl_3$. Starred signals are assigned to residual solvent.

Synthesis of P3OT-PFB-Cou$_{343}$:

Prepared according to conditions A of typical S_NAr procedure using Cou$_{343}$-SH at RT for 48 hours.

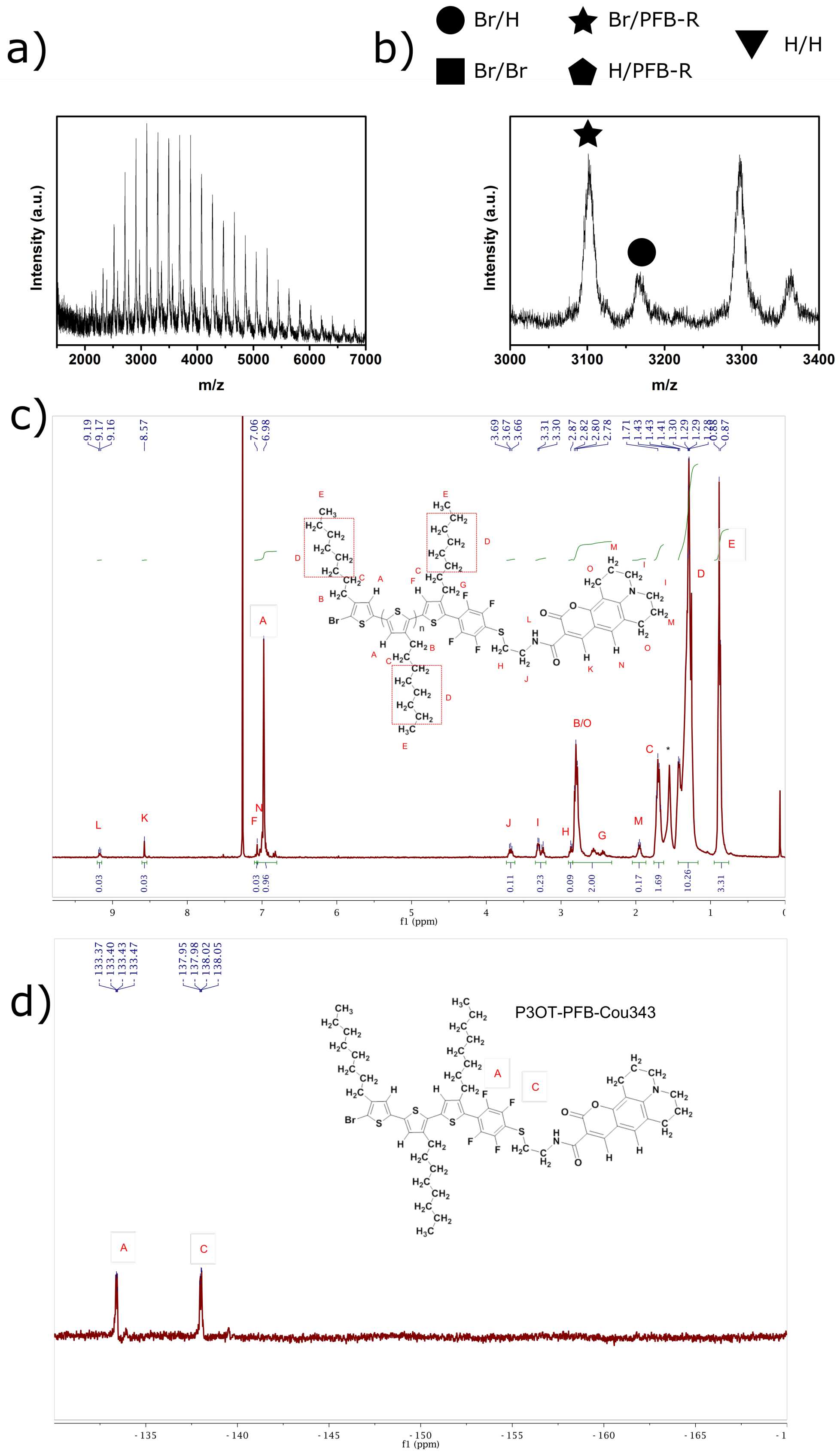

Figure S18. a) MALDI-TOF spectrum of P3OT-PFB-Cou_{343}. b) Selected region of MALDI-TOF spectrum with signals assigned, where R = -S-Cou_{343}. c) and d) are respectively ^{1}H and ^{19}F NMR of P3OT-PFB-Cou_{343} in $CDCl_3$. Starred signals are assigned to residual solvent.

Synthesis of P3OT-PFB-Biotin:

Prepared according to conditions A of typical S_NAr procedure using Biotin-SH at 70 °C for 12 hours.

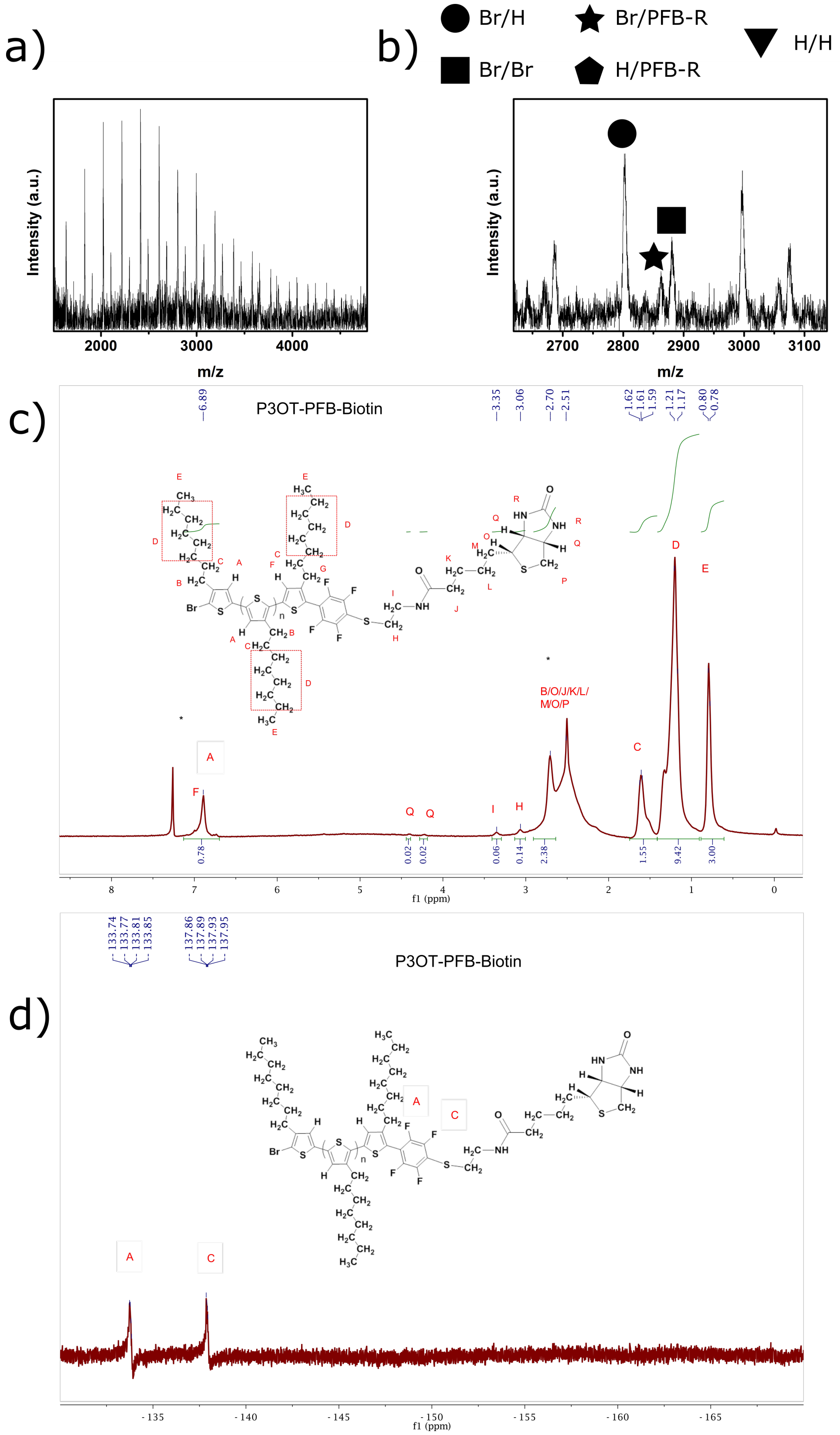

Figure S19. a) MALDI-TOF spectrum of P3OT-PFB-Biotin. b) Selected region of MALDI-TOF spectrum with signals assigned, where R = -S-Biotin. c) and d) are respectively ^{1}H and ^{19}F NMR of P3OT-PFB-Biotin in $CDCl_3$/DMSO-d^6(1:1). Starred signals are assigned to residual solvent.

Synthesis of P3OT-PFB-3MPTS:

Prepared according to conditions A of typical S_NAr procedure using 3MPTS at 70 °C for 12 hours.

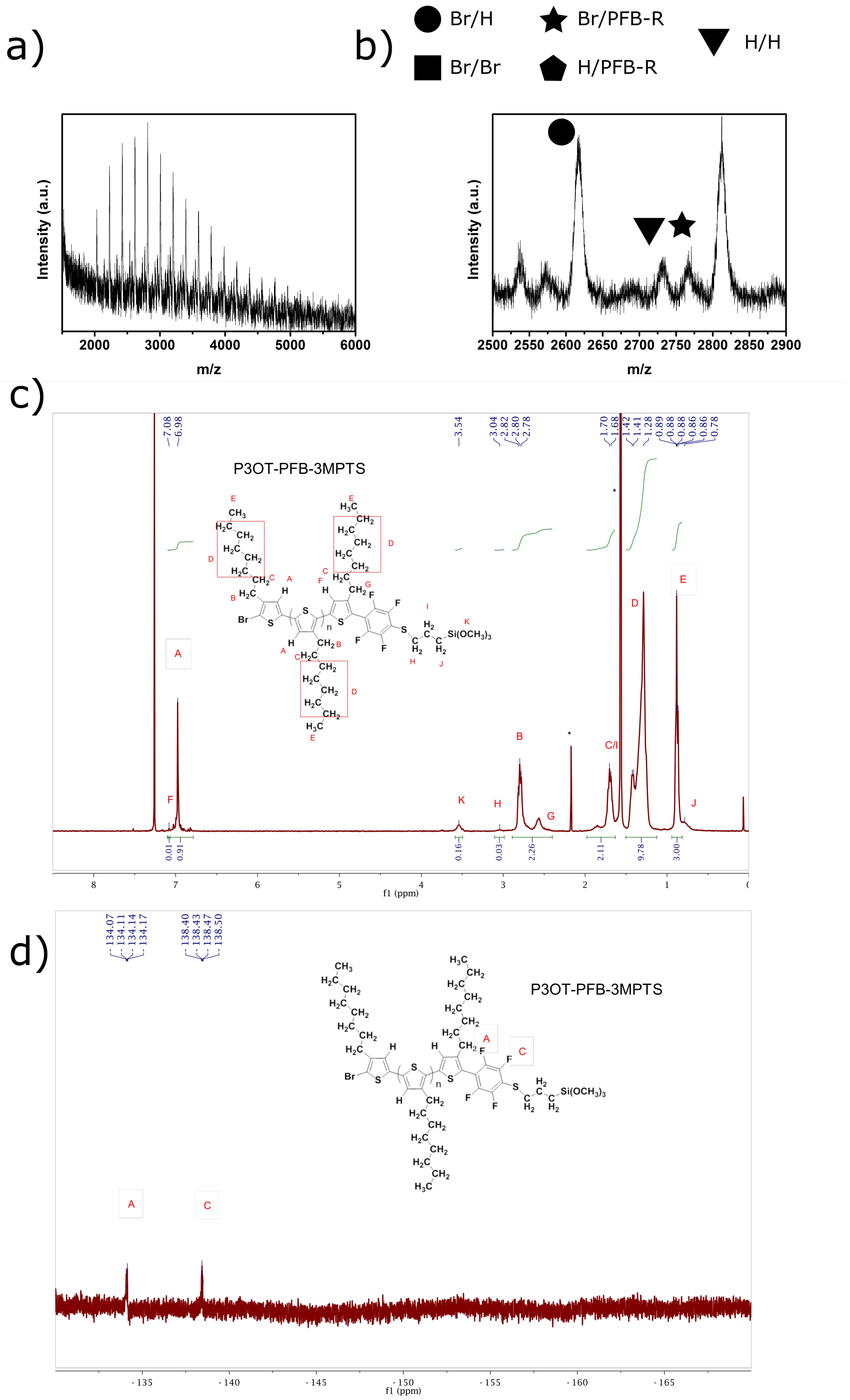

Figure S20. a) MALDI-TOF spectrum of P3OT-PFB-3MPTS. b) Selected region of MALDI-TOF spectrum with signals assigned, where R = -3MPTS. c) and d) are respectively ^{1}H and ^{19}F NMR of P3OT-PFB-3MPTS in $CDCl_3$. Starred signals are assigned to residual solvent.

Synthesis of P3EPT-PFB-SC$_{12}$H$_{25}$:

Prepared according to conditions D of typical S$_N$Ar procedure using dodecanethiol at RT for 40 minutes.

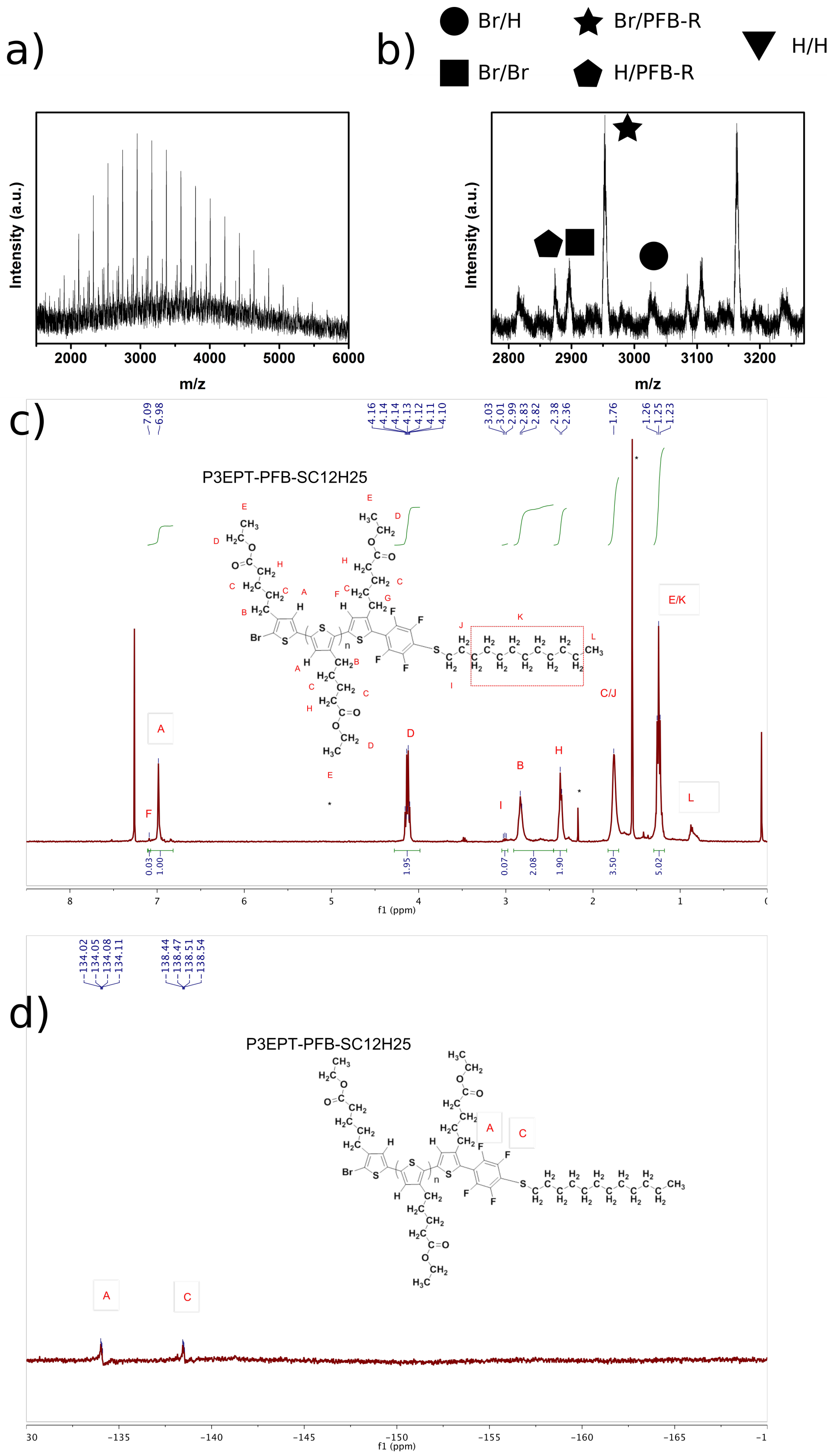

Figure S21. a) MALDI-TOF spectrum of P3EPT-PFB-$SC_{12}H_{25}$. b) Selected region of MALDI-TOF spectrum with signals assigned, where R = -$SC_{12}H_{25}$. c) and d) are respectively ^{1}H and ^{19}F NMR of P3EPT-PFB-$SC_{12}H_{25}$ in $CDCl_3$. Starred signals are assigned to residual solvent.

Synthesis of P3EPT-PFB-ODT:

Prepared according to conditions A of typical S_NAr procedure using 1,8-octanedithiol at 70 °C for 12h. Reaction was carried out under Ar. Polymer is precipitated in methanol, filtered and immediately washed with hot hexane to remove excess dithiol which otherwise polymerises upon oxidation.

Oxidation/reduction experiment of P3EPT-PFB-ODT:

P3EPT-PFB-ODT (10 mg, 0.005 mmol), triethylamine (10 uL, 0.072 mmol) and CuCl (10mg, 0.1 mmol) were dissolved in chlorobenzene (0.5 mL) and stirred at 50 °C for 12h. The reaction mixture was washed with water, and dried over $MgSO_4$ to give the oxidised disulfide product as demonstrated in the GPC elugram in the main text. The disulphide was reduced again by adding DL-dithiothreitol to the chlorobenzene solution and heating to 70 °C, adding more DL-dithiothreitol until the reduction was complete as evidenced by GPC analysis.

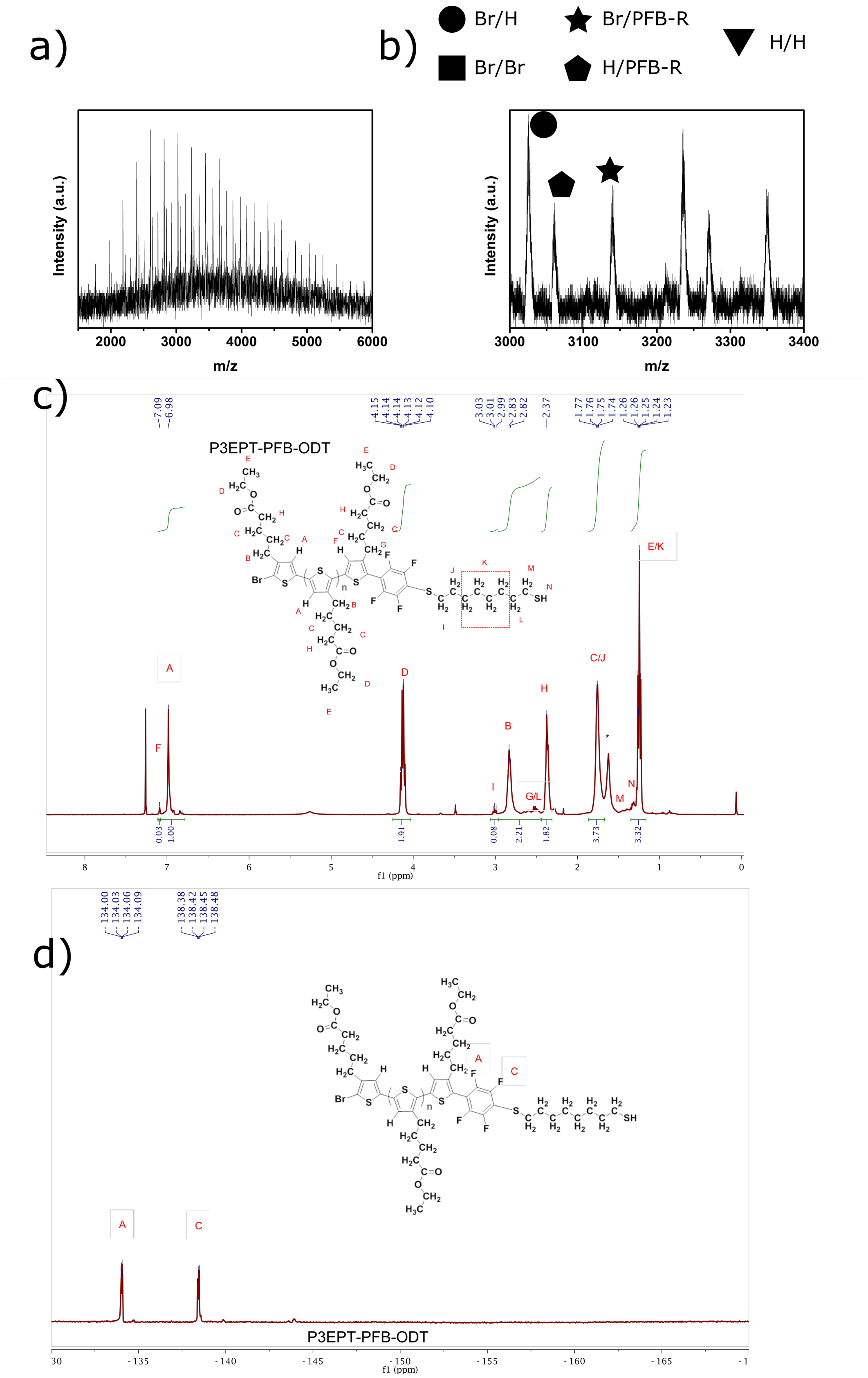

Figure S22. a) MALDI-TOF spectrum of P3EPT-PFB-ODT. b) Selected region of MALDI-TOF spectrum with signals assigned, where R = $-SC_8H_{16}SH$. c) and d) are respectively ^1H and ^{19}F NMR of P3EPT-PFB-ODT in $CDCl_3$. Starred signals are assigned to residual solvent.

Synthesis of P3EPT-PFB-3ODT:

Prepared according to conditions D of typical S_NAr procedure using 1,8-octanedithiol at RT for 2h. Reaction was carried out under Ar.

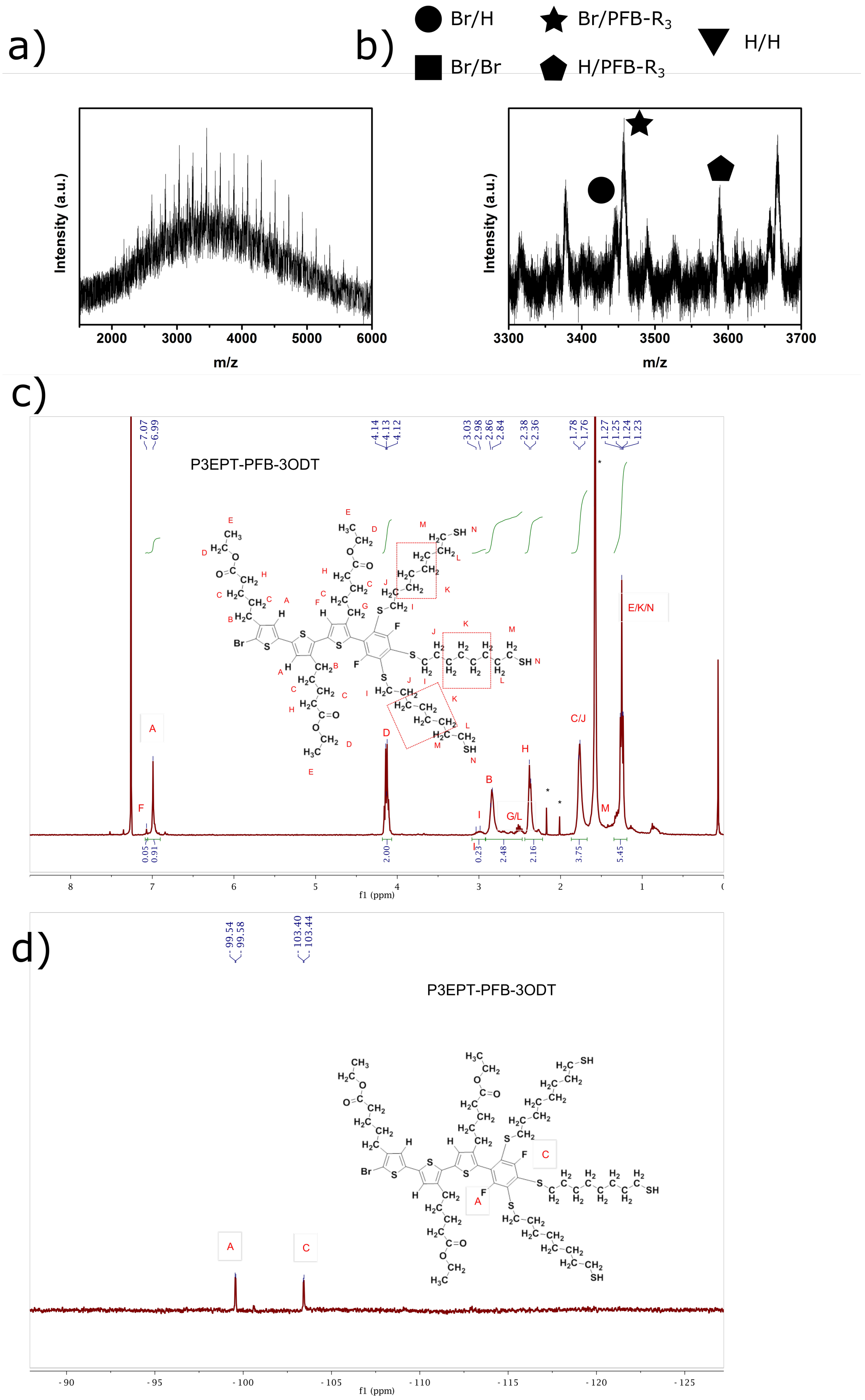

Figure S23. a) MALDI-TOF spectrum of P3EPT-PFB-3ODT. b) Selected region of MALDI-TOF spectrum with signals assigned, where R = -$SC_8H_{16}SH$. c) and d) are respectively ^{1}H and ^{19}F NMR of P3EPT-PFB-3ODT in $CDCl_3$. Starred signals are assigned to residual solvent.

Synthesis of P3EPT-*b*-PEG:

Prepared according to conditions D of typical S_NAr procedure using mPEG-SH (1 eq.) at RT for 12h. Reaction was carried out under Ar. On workup, reaction mixture was evaporated and titurated with chloroform. The chloroform was then evaporated yielding the product as a red solid.

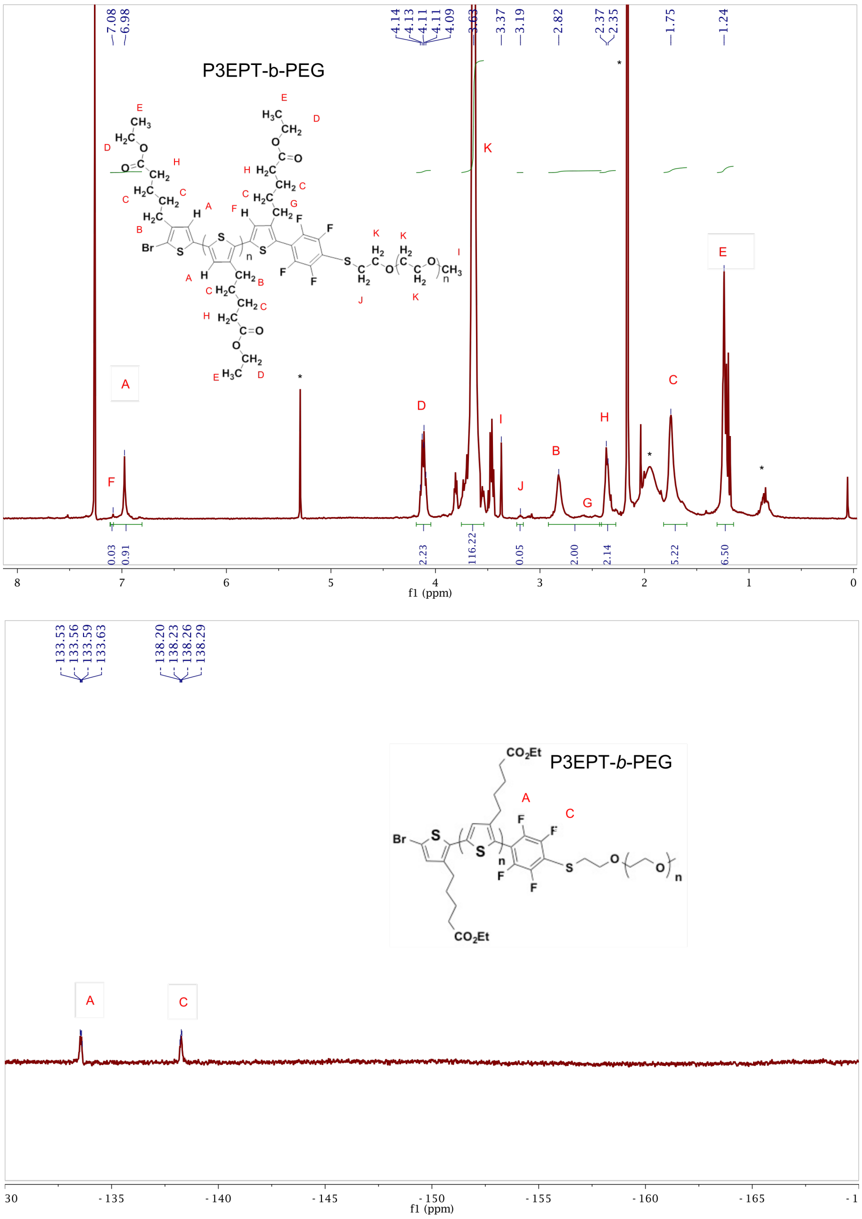

Figure S24. ^{1}H (top) and ^{19}F (bottom) NMR of P3EPT-*b*-PEG in $CDCl_3$. Starred signals are assigned to residual solvent.

Synthesis of P3APT-PFB-cys:

Prepared according to conditions D of typical S_NAr procedure (with H_2O added as cosolvent) from P3APT-PFB and cysteine at RT for 7h. Reaction mixture was then acidified with dilute HCl and filtered, and washed with acetonitrile.

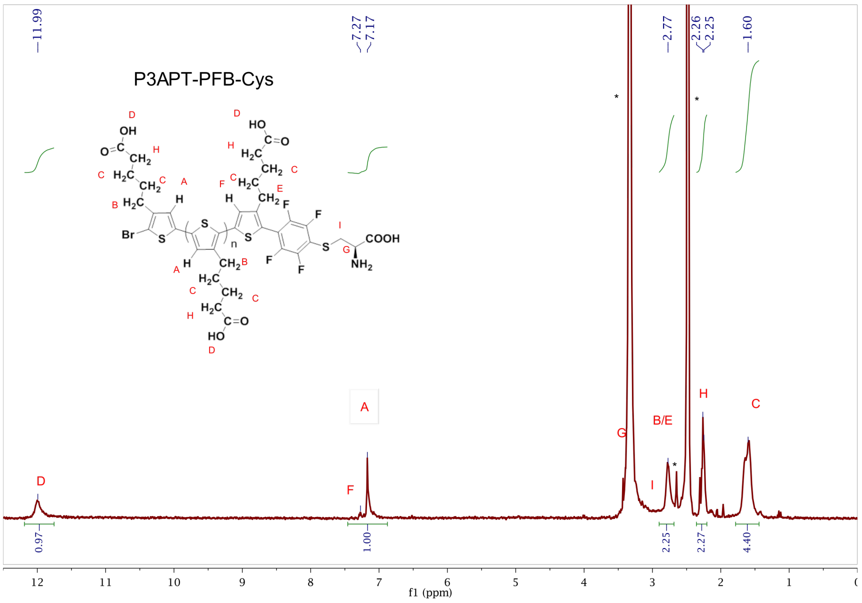

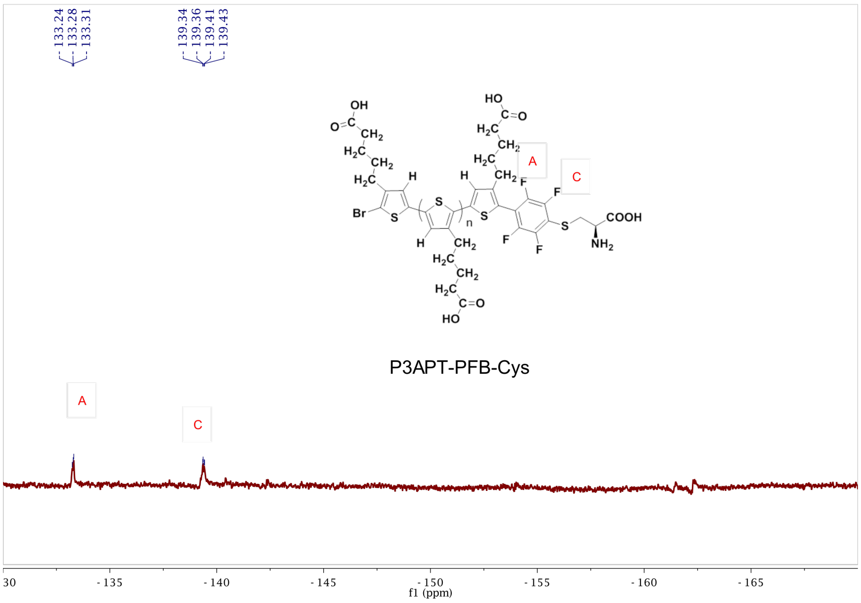

Figure S25. ^{1}H (top) and ^{19}F NMR (bottom) of P3APT-PFB-Cys in DMSO-d^{6}. Starred signals are assigned to residual solvent.

References

(1) Kim, S. H.; Kim, J. G. *Bull. Korean Chem. Soc.* **2009**, *30*, 2283.

(2) Roling, O.; Wendeln, C.; Kauscher, U.; Seelheim, P.; Galla, H. J.; Ravoo, B. J. *Langmuir* **2013**, *29*, 10174.

(3) Krasovskiy, A.; Knochel, P. *Synthesis (Stuttg).* **2006**, *2006*, 0890.
